# Guide RNA Repertoires in the Main Lineages of *Trypanosoma cruzi*: High Diversity and Variable Redundancy Among Strains

**DOI:** 10.3389/fcimb.2021.663416

**Published:** 2021-05-31

**Authors:** Fanny Rusman, Noelia Floridia-Yapur, Nicolás Tomasini, Patricio Diosque

**Affiliations:** Unidad de Epidemiología Molecular (UEM), Instituto de Patología Experimental, Universidad Nacional de Salta-CONICET, Salta, Argentina

**Keywords:** Chagas disease, kinetoplastids, minicircles, RNA editing, DTU

## Abstract

*Trypanosoma cruzi*, as other kinetoplastids, has a complex mechanism of editing of mitochondrial mRNAs that requires guide RNAs (gRNAs) coded in DNA minicircles in the kinetoplast. There are many variations on this mechanism among species. mRNA editing and gRNA repertoires are almost unknown in *T. cruzi*. Here, gRNAs were inferred based on deep-sequenced minicircle hypervariable regions (mHVRs) and editing cascades were rebuilt in strains belonging to the six main *T. cruzi* lineages. Inferred gRNAs were clustered according to their sequence similarity to constitute gRNA classes. Extreme diversity of gRNA classes was observed, which implied highly divergent gRNA repertoires among different lineages, even within some lineages. In addition, a variable gRNA class redundancy (i.e., different gRNA classes editing the same mRNA region) was detected among strains. Some strains had upon four times more gRNA classes than others. Such variations in redundancy affected gRNA classes of all mRNAs in a concerted way, i.e., there are correlated variations in the number of gRNAs classes editing each mRNA. Interestingly, cascades were incomplete for components of the respiratory complex I in several strains. Finally, gRNA classes of different strains may potentially edit mitochondrial mRNAs from other lineages in the same way as they edit their own mitochondrial mRNAs, which is a prerequisite for biparental inheritance of minicircle in hybrids. We propose that genetic exchange and biparental inheritance of minicircles combined with minicircle drift due to (partial) random segregation of minicircles during kDNA replication is a suitable hypothesis to explain the divergences among strains and the high levels of gRNA redundancy in some strains. In addition, our results support that the complex I may not be required in some stages in the life cycle as previously shown and that linkage (in the same minicircle) of gRNAs that edit different mRNAs may prevent gRNA class lost in such stage.

## Introduction

Kinetoplastids are a phylogenetic group of flagellate protozoa that include the genus *Leishmania* and *Trypanosoma*, with three species causing neglected diseases: Leishmaniasis, Chagas disease and sleeping sickness. Interestingly, kinetoplastids have a single large mitochondrion with a disk shape structure called kinetoplast. This structure has a complex network of concatenated DNA rings (Cavalcanti and de Souza, 2018). Two different kinds of DNA rings are contained in such network: maxicircles and minicircles. Maxicircles are large molecules (20-30 kb) that appear in a few dozens of almost identical copies. They code for two mitochondrial ribosomal subunits and eighteen mitochondrial proteins (most of them participating in the respiratory chain) (Simpson et al., 1987; Ruvalcaba-Trejo and Sturm, 2011; Lin et al., 2015). Several of such genes require post-transcriptional modifications in their pre-mRNA to generate functional open reading frames. The process is known as mRNA editing and it is made by inserting or deleting uridines (U) (Benne et al., 1986; Koslowsky et al., 1991; Stuart, 1993; Kim et al., 1994; Simpson et al., 1996; Hajduk et al., 1997; Hajduk and Ochsenreiter, 2010; Ammerman et al., 2012; Aphasizheva et al., 2016). Some mRNAs require extensive editing, a phenomenon known as pan-editing [e.g. more than six hundred edited bases to generate the cytochrome c oxidase subunit III (COIII) open reading frame in *T. brucei* (Feagin et al., 1988)]. Instead, other mRNAs are just partially or non-edited.

Uridine insertion/deletion is directed by short RNAs called guide RNAs (gRNAs) and it works as a cascade of steps. Basically, the first gRNA binds the pre-mRNA at a specific and complementary site (non-canonic G::U base pairing is allowed) in the 3’ extreme of the mRNA. Then, it determines the editing of the adjacent sites at the 5’ based on mismatches (Estevez and Simpson, 1999). This modified region is recognized by another gRNA which drives the editing of the next adjacent region, and successively up to generate the initial codon in the 5’ end. If gRNAs that edit a region are lost, the editing is stopped, and no translation is made (Estevez and Simpson, 1999; Lukes et al., 2005). Most editing cascades have been identified for *T. brucei* (Ochsenreiter et al., 2007; Koslowsky et al., 2014; Kirby et al., 2016), *T. vivax* (Greif et al., 2015) and *L. tarentolae* (Simpson et al., 2015). Different evolutionary hypotheses have been proposed to explain the origin and persistence of this expensive editing system, although no consensus has been achieved yet (Gray et al., 2010; Speijer, 2010; Flegontov et al., 2011).

Guide RNAs are coded in the minicircles. In *T. cruzi* there are about 20-30 thousand minicircles per parasite, 1.4 kb each one. Each minicircle contains four hypervariable regions (mHVRs) interspersed by four conserved regions located 90° apart each other. Every mHVR potentially encodes one gRNA (Degrave et al., 1988; Velazquez et al., 2008).

Most of our knowledge on mitochondrial mRNA editing in trypanosomatids comes from studies in *T. brucei* and *L. tarentolae*, while little is known about editing in *T. cruzi* (Thomas et al., 2007). This parasite is a very diverse species and seven different lineages (also called Discrete Typing Units or simply DTUs) have been described: the six main lineages TcI-TcVI (Zingales et al., 2009) plus TcBat (Marcili et al., 2009; Pinto et al., 2012; Lima et al., 2015). Strains belonging to the six main lineages of *T. cruzi* can be classified in three different mitochondrial clades according to their maxicircle sequences (Machado and Ayala, 2001; de Freitas et al., 2006; Westenberger et al., 2006; Ruvalcaba-Trejo and Sturm, 2011).

Recently, we deep-sequenced the mHVRs from 9 strains of this parasite at millions of paired-end reads (Rusman et al., 2019). The number of mHVR sequence clusters (according to pairwise identity) was quite different among most strains and the different DTUs shared few mHVR clusters. These results suggest that different DTUs, and even strains, may have different gRNA repertoires. In addition, such differences in the number of mHVR clusters may imply redundant gRNAs (gRNAs with relatively different sequences that edit the same region) or mHVRs not encoding gRNAs. We proposed that minicircle inheritance is biparental in hybrid DTUs (Rusman et al., 2019) and hybrid strains (Rusman et al., 2020) despite the uniparental inheritance of maxicircles (Tomasini, 2018). This scenario opens the question on how mixing minicircles from different parents could still lead to correct editing of mitochondrial mRNAs, since maxicircles are inherited from one parental. The mHVR sequences have previously been used to identify gRNAs and to build editing cascades by comparing them with edited mRNAs (Thomas et al., 2007; Simpson et al., 2015). Here, based on mHVR reads previously obtained, an algorithm was developed to identify and cluster gRNA sequences from big datasets. The aims of this study were to describe and compare editing cascades for representative strains of the *T. cruzi* diversity, to address differences in mRNA editing and to infer how gRNA repertoires evolve.

## Material and Methods

### mHVR Sequences

The strains analyzed in this study are listed in **Table 1**. The paired-end mHVR amplicon sequences were obtained by Rusman et al. (2019). Reads are available at the Sequence Read Archive (SRA) under the BioProject ID: PRJNA514922. Raw sequence reads for all samples were pre-processed. First, reads were quality filtered using the pair-end mode of Trimmomatic v0.36 (Bolger et al., 2014). Posteriorly, the preserved paired reads were merged into consensus sequences with their associated quality score and chimeras were removed using LeeHom software with default parameters (Renaud et al., 2014).

**Table 1:** Datasets used in this study.

Accession	Strain	DTU	Mitochondrial clade	Origin	Host
SRX5245771	PalDa20cl3	TcI	A	El Palmar, Argentina	*Didelphis albiventris*
SRX5245770	TEV55cl1	TcI	A	Tres Estacas, Argentina	*Triatoma infestans*
SRX5245773	Esmeraldo	TcII	C	Sao Felipe, Brazil	*Homo sapiens*
SRX5245772	TU18cl93	TcII	C	Potosí, Bolivia	*Triatoma infestans*
SRX5245767	X109/2	TcIII	B	Makthlawaiya, Paraguay	*Canis familiaris*
SRX5245766	CANIIIcl1	TcIV	B	Belém, Brazil	*Homo sapiens*
SRX5245769	MNcl2	TcV	B	Región IV, Chile	*Homo Sapiens*
SRX5245768	LL014R1	TcV	B	Las Leonas, Argentina	*Triatoma infestans*
SRX5245774	L015P68R0cl4	TcVI	B	Las Leonas, Argentina	*Canis familiaris*

### Mitochondrial mRNA Estimation

Predicted edited mRNA sequences for strains Sylvio (mitochondrial clade A, TcI), CL Brener (mitochondrial clade B, TcVI) and Esmeraldo (mitochondrial clade C, TcII) (Ruvalcaba-Trejo and Sturm, 2011) were generated by manually inserting or deleting uridines (U’s) into the unedited sequences of their genes as proposed by (Greif et al., 2015). Briefly, unedited sequences corresponding to Sylvio (FJ203996.1), CL Brener (DQ343645.1) and Esmeraldo (DQ343646.1) obtained from the NCBI database were aligned using MEGA v7 software (Kumar et al., 2016). Then, the alignment was manually manipulated by inserting or deleting uridines (U’s) at specific sites into the unedited sequences following the editing patterns of the corresponding *T. brucei* and *T. vivax* edited mRNAs, meanwhile the resulting amino acid sequence was preserved. Predicted sequences were generated for *mitochondrial unidentified reading frame 2* (MURF2), *NADH dehydrogenase subunit 3* (ND3) and *C-rich region 4* (CR4) following the known edited mRNAs for *T. vivax* and *T. brucei* (**Supplementary File 1**). On the other hand, sequence data of predicted edited mRNAs for the following genes were obtained from Ruvalcaba-Trejo et al. (Ruvalcaba-Trejo and Sturm, 2011) for the same three *T. cruzi* strains: ATPase6, *C-rich region 3* (CR3), *cytochrome b* (CyB), *ribosomal protein S12* (RPS12), *NADH dehydrogenase subunit 7* (ND7), *NADH dehydrogenase subunit 8* (ND8) and *NADH dehydrogenase subunit 9* (ND9). CL Brener *cytochrome oxidase subunit 3* (COIII) mRNA was downloaded from GenBank (accession number: EF058194.1). In addition, Sylvio and Esmeraldo COIII edited mRNA sequences were manually generated (**Supplementary File 1**). Additional maxicircle sequences described by Reis-Cunha et al. 2018) were downloaded from GenBank, manually edited, and also used for gRNA search. Predicting gRNAs based on such sequences (shown in **Table S1**) had similar results to the obtained by using Sylvio, Esmeraldo or CL-Brener edited mRNAs.

### gRNA Detection

An algorithm using c++ and the SeqAn library (Reinert et al., 2017) was built to detect potential gRNA in the mHVR reads. First, a hash-table was built from the reads by storing position of all the possible k-mers (seeds) of 15 bases. However, due to non-canonical base pair a hash-table was used considering just purines (R) and pyrimidines (Y) instead the four bases. Although C::A pairing is allowed with this approach, it is considered a mismatch in the following steps. An algorithm scheme is provided in **Figure S1**. The hash table only allows to reduce the search time in the next steps. The mRNA sequence for each edited gene was used to identify gRNAs. The estimated edited mRNA of the corresponding mitochondrial clade was used to infer gRNA for each strain. First, a window of 15 bases was selected at the 5’ end in the mRNA and the reverse complement of the purine/pyrimidine pattern was used to determine which mHVRs contains a potential gRNA by using the hash-table. Then, the potential site was evaluated considering canonical and non-canonical base pairing (G::U) with the mRNA and allowing only one mismatch. Finally, the pairing is extended to both sides using an x-drop extension allowing one mismatch. Mismatches were allowed considering some flexibility in editing and considering that mRNAs are estimated and minimal errors in estimations are possible (Thomas et al., 2007). A filter was then applied to discard the predicted gRNA if it has fewer bases than the parameter f. This parameter is the minimum number of bases that a gRNA candidate must have to be accepted. This parameter is required because short sequences with complementarity to mRNAs are expected by simple random. Then, the window is moved over the mRNA one base at a time and the process is repeated until the entire mRNA is scanned. Different values for f were evaluated in order to discard sequences that are not gRNAs. In a first approach, gRNAs with <40 bases were discarded (f = 40). This filter was too stringent but allowed to identify the most probable regions of the mHVR that contain the gRNA, that is the gRNA cassette. Then, the filter was lowered looking for hits outside the cassette and the minimum filter that had as maximum as 5% of the gRNA hits out of the cassette was selected (f = 30) (**Figure S3**). Alternatively, other approaches for gRNA detection were evaluated. The first one was based on (Koslowsky et al., 2014). Briefly, after looking for 15 bp seeds, the algorithm scored canonical (score = 2) and non-canonical (score = 1) base pairings and gRNAs with scores <45 were discarded. The second approach was based on (Cooper et al., 2019) by looking for gRNAs with 6 bp-anchors (canonical base pairing) in the 5’ end and then discarding gRNAs with less than 25 bp (canonical plus non-canonical base pairing). Both approaches were too stringent, they recovered reduced gRNA coverage in ATPase 6 mRNA and a smaller number of gRNAs than using the above approach f = 40 (**Figure S1**). Consequently, f = 30 was established given its higher sensitivity than f = 40, and a reasonable rate of gRNA hits out of the cassette. The c++ source code for gRNA detection in mHVRs and example datasets are stored in https://osf.io/kn34z/.

### gRNA Clustering Analyses

Identified gRNAs were clustered according to sequence similarity and overlapping in the edited-mRNA target region. A SWARM-like algorithm (Mahe et al., 2014) was implemented to cluster gRNAs. SWARM is a fast unsupervised (*de novo*) single-linkage-clustering algorithm. An algorithm scheme is provided in **Figure S2**. The modified algorithm also included an overlapping threshold (*o*) in addition to a threshold of the maximum number of base mismatches between sequences (*d*). Basically, the algorithm selects one gRNA as a cluster seed and then it iteratively looks for gRNAs with a number of mismatches lower than *d* and an overlapping > *o* and adds them to the cluster. Then, it iteratively looks for unclustered gRNAs with less than *d* mismatches and overlapping > *o* to every gRNA previously included in the cluster. When no new gRNAs can be added to the cluster a new cluster is seeded using a gRNA that was not previously clustered. The process is iterated until the all gRNAs were joined to a cluster. *o* = 0.7 and *d* = 4 were implemented. This is a relaxed threshold to cluster moderately different gRNAs in the same cluster considering divergency in such kind of sequences. Considering the overlapping region of two gRNAs of ~30-50bp, *d* = 4 implies 85-92% pairwise similarity. Similar percentages were used to cluster mHVRs in (Rusman et al., 2019). The second phase of breakage in the original SWARM algorithm was not required (Mahe et al., 2014) because of the high distances and reduced overlapping among clusters. Identified gRNA clusters based on SWARM were termed as gRNA classes here. Finally, a consensus gRNA was built for every gRNA class to represent editing cascades. In order to fast calculation, the consensus gRNA was built using a base-by-base majority-rule criterium (i.e. the most abundant base in each position of gRNA alignment was used to build the consensus gRNA). Because artifactual gRNA may be generated by this method the algorithm also calculated the number of gRNAs sharing such sequence and informed it in the output file to manual inspection. The consensus gRNAs were graphed aligned to the edited mRNA and the cluster abundance was represented with a color scale.

In order to assess linkage disequilibrium as a measure of genetic structuring among strains, the index of association (Ia) and the proportion of compatible pairs of loci with strict clonality (PrCompat) were calculated. Briefly, gRNA classes were coded as present (>20 reads) or absent (≥20 reads) emulating RFLP pattern data for each strain and the coded matrix was analyzed as haploid data in Mulilocus v1.3 (Agapow and Burt, 2001) with 10,000 iterations to calculate statistical significance.

A subset of gRNA classes was selected to predict secondary structure. Briefly, gRNA classes with more than 200 reads (high abundance), with more than 90% of the reads being identical to the consensus sequence of the class and with at least 40 bases were selected. The selected class representative gRNAs were analyzed using RNAstructure software (Reuter and Mathews, 2010). The maximum accuracy structures were calculated using default parameters but considering a temperature of 27°C (optimal to temperature for epimastigotes and previously used in *T. brucei* analysis of gRNA secondary structure) (Schmid et al., 1995).

### mHVR Clustering and Comparison With gRNA Classes

mHVR reads were processed and clustered according to (Rusman et al., 2019) using a UCLUST algorithm with a threshold of 85% of identity for cluster definition. Clustering at 85% was previously used to define mHVR clusters (Rusman et al., 2019) and such clusters were used to determine relationships between mHVR clusters and gRNA classes. Briefly, a c++ algorithm was designed to add a string with the mHVR cluster ID to each read name in the raw fastq file. Then, this fastq file was used to determine gRNAs using the above-described algorithm. This way, each identified gRNA could be assigned to an mHVR cluster. After clustering gRNAs in classes using the SWARM algorithm, it was evaluated whether each mHVR cluster coded none, one or more than one gRNA. Around 9.5% of the clusters corresponded to two gRNA classes in the same mHVR cluster. Although it is expected none or just one gRNA in an mHVR, such possible method inaccuracy was allowed in order to gain sensitivity to obtain the maximum coverage on mRNA editing cascades.

## Results

### Fast Evolutionary Changes in gRNA Repertoires

**Figure 1:**
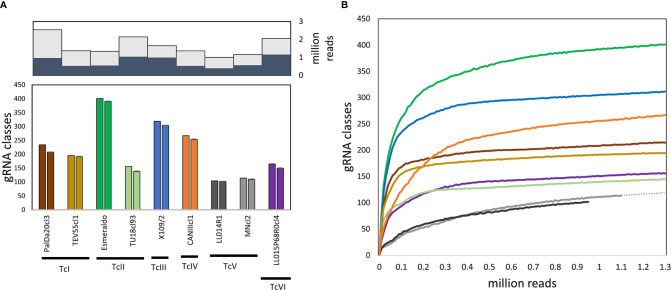
Variable gRNA diversity among strains. **(A)** Top, mHVR sequences with gRNA hits (dark grey) related to the number of analyzed mHVR sequences (light gray); bottom, number of gRNA classes (>20 reads) using a SWARM-like algorithm and detected for each strain (left column for each strain) and the average number of gRNA classes after down-sampling one-hundred times the number of paired-end reads to 950,000 (right column for each strain) with standard deviation bars. **(B)** Rarefaction curves for the number of gRNA classes (>20 reads) for each strain (solid lines, the color reference is according to **(A)**. Dotted line is an extrapolation for MNcl2 based on a linear regression using last ten samples showing that increasing the sequencing depth will not significantly increase the number of detected gRNA classes.

**Figure 2:**
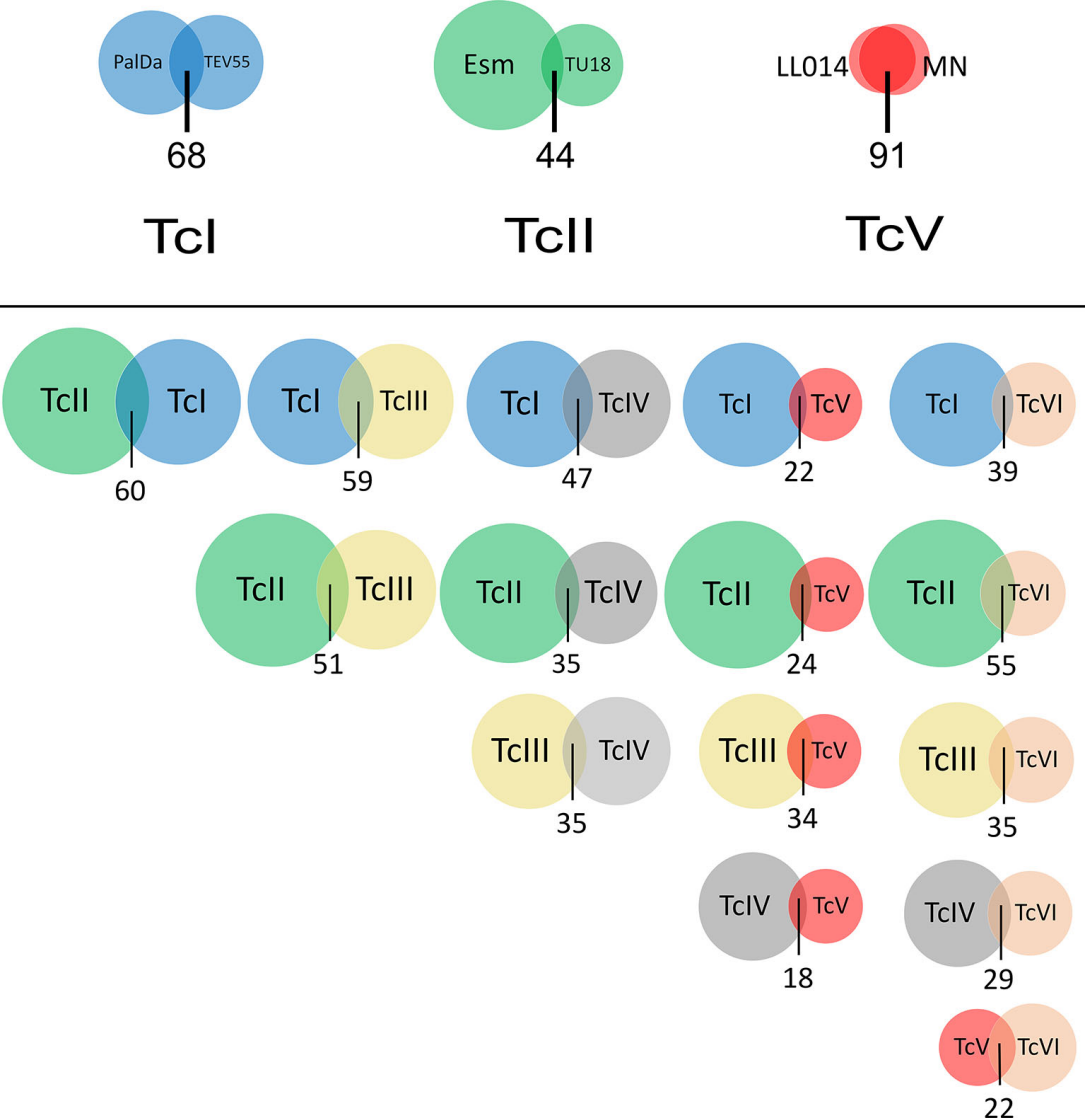
Venn diagrams showing overlapping gRNA repertoires between strains of the same DTU and between DTUs. Circle size represents the number of gRNA classes identified in a strain (above) or in a DTU (below). The overlapping area among circles indicates gRNA classes with more than 20 reads that are shared between strains or DTUs. The number below circles represents the percentage of shared gRNA classes. PalDa=PalDa20cl3, TEV=TEV55cl1, Esm=Esmeraldo, X109=X109/2, Tu18=Tu18cl93, MN=MNcl2 and LL014=LL014R1.

Guide RNAs coded in mHVRs were inferred based on the available sequences or predicted edited mRNAs of *T. cruzi*. A total of 7,476,003 gRNAs were detected for nine *T. cruzi* strains belonging to the six DTUs. The percentage of detected gRNAs from the total number of mHVR reads for each studied strain varied from 38% to 60% (**Figure 1A**), suggesting that some mHVRs would not encode gRNAs. Posteriorly, the gRNAs were clustered in gRNA classes according to the editing region and their sequence similarity (**Supplementary File 2**). A total of 1334 gRNA classes were identified (every class with >20 reads). The number of gRNA classes *per* strain was variable ranging from 104 (LL014R1-TcV) to 401 (Esmeraldo-TcII). Particularly, TcV strains had up to three or four times fewer gRNA classes than other DTUs (**Figure 1**). The number of gRNA classes was also variable within DTUs (**Figure 1** and **Figure 2**) suggesting fast changes in gRNA repertoires and probably gRNA redundancy (i.e., different gRNA classes editing the same mRNA region). Rarefactions of each dataset showed that these differences between strains are not the effect of different sequencing depths (**Figure 1B**).

Just 22.8% of the whole identified gRNA classes in all strains were shared among two or more of them and only four gRNA classes were shared among all strains (sequences provided in **Supplementary File 2**) revealing high divergence. However, the percentage of shared gRNA classes between strains of the same DTU was variable (**Figure 2**). TcI strains shared a relatively low number of gRNA classes (19.2%) despite that the studied strains are very close phylogenetically (according to nuclear genes) (Schmid et al., 1995) and they were isolated in the same geographical area and time (Diosque et al., 2003). In addition, TcII strains shared even few gRNA classes (9.1%) (**Figure 2**). Instead, TcV strains (LL014R1 and MNcl2) shared a high proportion of gRNA classes (73.8%) despite they were isolated in geographically distant places and with a difference of around 30 years between the isolation dates. All these results suggest significant changes in gRNA repertoires in relatively short evolutionary times. In addition, linkage disequilibrium was analyzed to address the structuring of such diversity. The index of association (Ia) was 49.3 and showed high significance (p < 0.001). Furthermore, the ratio of pairs of gRNA classes compatible (PrCompat) with unmixed gRNA classes was 0.98 suggesting structuring of diversity among strains. Finally, it was addressed whether probable predicted secondary structures are common in such gRNA sequence diversity. A subset of 89 gRNA classes defined by a stringent criterion (see Material and methods) was studied. Although variable structures were observed (**Supplementary File 4**), 70.7% of the gRNAs have a stem region intercalated by one or two internal loops (or less frequently bulges) and ending in a hairpin loop (**Figure S4**). Only 26% of the structures have a double hairpin (**Figure S4**).

### Silent mHVRs

In a previous work, entire mHVR sequences were clustered according to sequence identity (Rusman et al., 2019). Here, the gRNA classes were assigned to mHVR clusters. First, most (97.1%) mHVR clusters of all strains had one of two states: (i) high proportion (> 75%) of the sequences in the mHVR cluster coding one gRNA class; or (ii) low proportion (< 10%) of sequences in the mHVR cluster coding a gRNA class (**Table S2**). Most exceptions to the rule were observed in TcV strains (**Table S1**). The term “silent” mHVR clusters was used for those in which less than 20 gRNAs were identified. Silent mHVR clusters were from 25% to 54% of the total mHVR clusters in the studied strains (**Table 2**). Curiously, strains with the lowest number of gRNA classes (TcV and TcVI strains) also had the lowest percentage of silent mHVR clusters (**Table 2**).

**Table 2:** Relationship between mHVR clusters and gRNA classes.

	TcI		TcII		TcIII	TcIV	TcV		TcVI
	PalDa20cl3	TEV55cl1	Esmeraldo	TU18cl93	X109/2	CANIIIcl1	LL014R1	MNcl2	LL015P68R0cl4
mHVR clusters^1^	324	234	347	151	373	149	72	71	108
Silent mHVR clusters^2^	47%	39%	42%	37%	43%	54%	32%	34%	25%

^1^mHVR clusters were defined in Rusman et al. (2019).

^2^mHVR clusters for which less than 20 gRNAs were detected.

**Figure 3:**
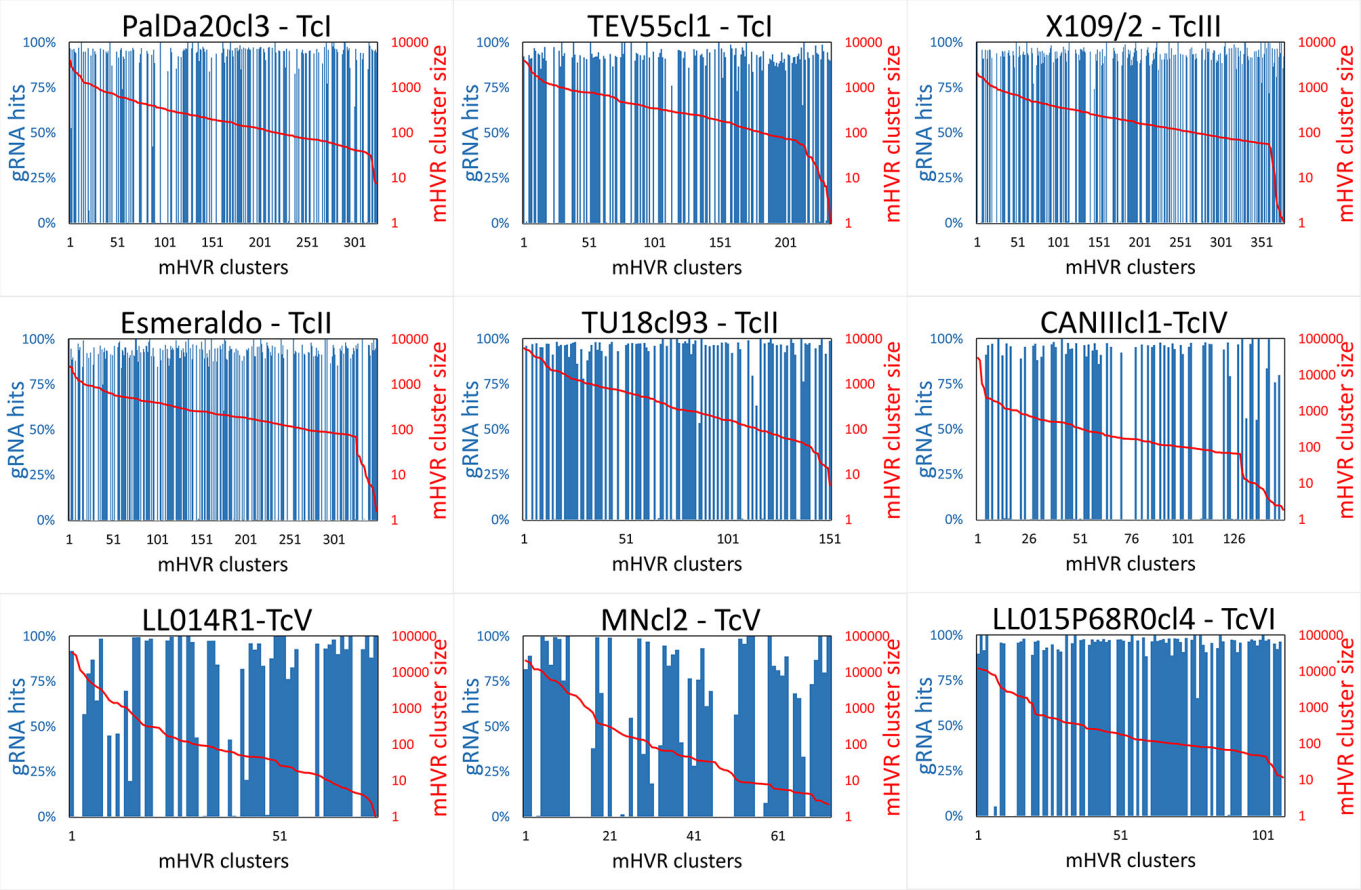
The number of mHVR sequences in a cluster is not related to the coding of gRNAs. mHVR clusters were graphed in decreasing order according to their size (mHVR cluster size is indicated with a solid redline). Each blue bar indicates the percentage of reads in the mHVR cluster in which a gRNA was identified (gRNA hits). Red color indicates percentage of reads in the mHVR cluster where gRNAs were not detected.

Furthermore, the analyses showed there is no clear relationship between mHVR cluster size and whether it encodes a gRNA or not since silent mHVR clusters were of quite different abundances (**Figure 3**).

### Concerted Changes in the Number of gRNA Classes

**Figure 4:**
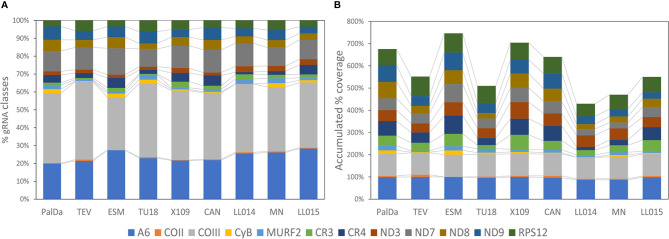
Balanced changes in gRNA repertoires of different strains and variable coverage for different mRNAs. **(A)** Relative percentage of the number of gRNA classes identified for each mRNA. **(B)** Accumulated coverage percentage for each mRNA by gRNAs. The coverage percentage for each mRNA is calculated as the percentage of the mRNA sequence that aligned with gRNA classes.

The gRNA classes identified for each strain were aligned with the edited mRNAs of the corresponding mitochondrial clade. The number of gRNA classes editing each gene for all strains is represented in **Figure 4** and **Table S3**. Most gRNA classes (more than 50%) edit ATPase 6 and COIII mRNAs in all strains (**Figure 4A**). Interestingly, the percentage of gRNA classes editing each mRNA was nearly conserved among strains (**Figure 4B**). This implies correlated variations in the number of gRNAs classes editing each mRNA (see correlation coefficients in **Table S4**). These results show that observed variations in the number of gRNA classes are balanced, i.e., if one strain decreased gRNA classes for one mRNA also decreased the number of gRNAs classes for editing other mRNAs. This would be an expected result whether each minicircle codes for different gRNAs, each one editing different mRNAs.

### Editing mRNA Cascades for ATPase6 and COIII

**Figure 5:**
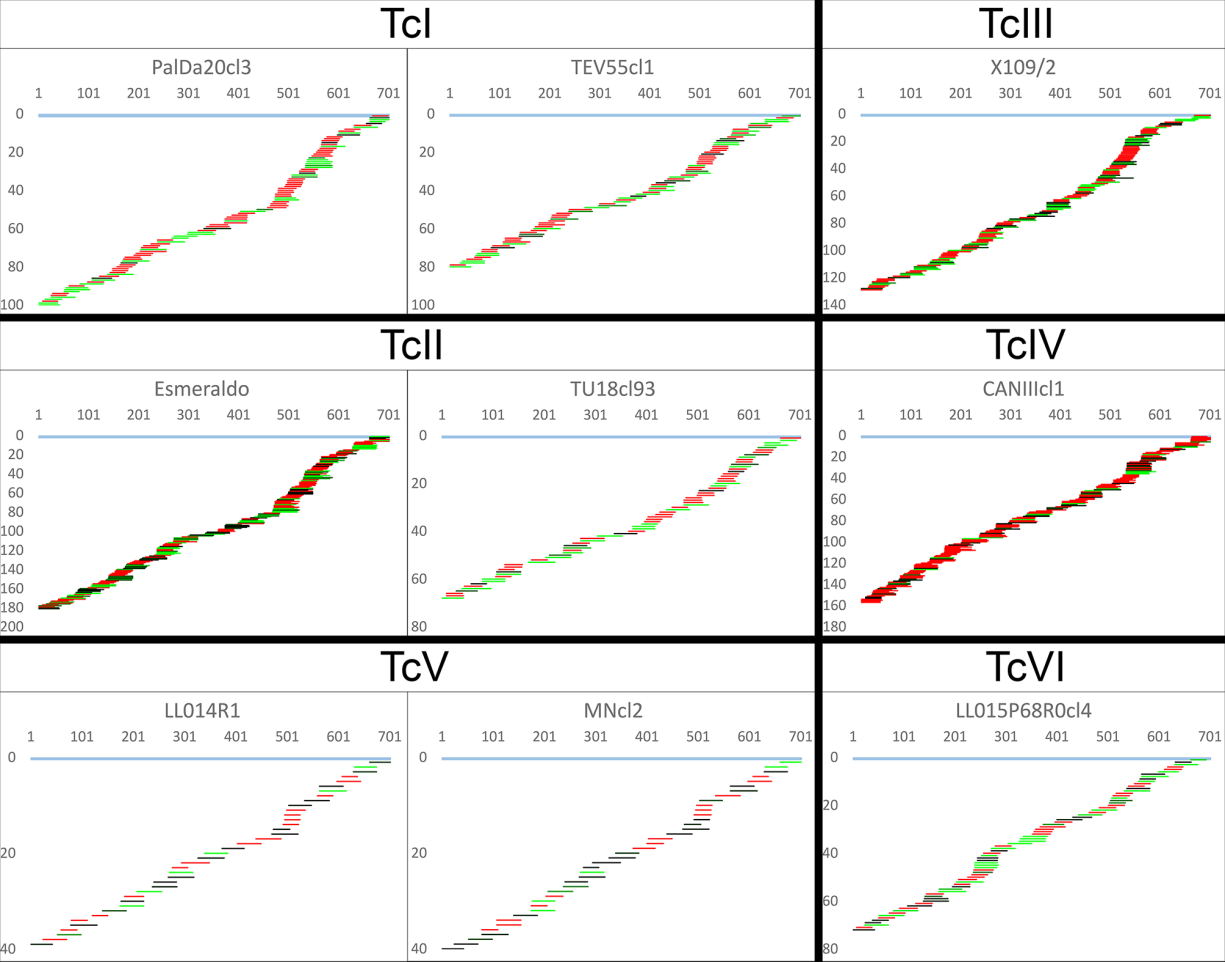
Canonical ATPase6 mRNA editing cascade inferred from mHVR reads. gRNA classes are shown aligned below the fully edited mRNA (light blue). The x-axis indicates mRNA position, and the y-axis indicates the accumulated number of gRNA classes until such mRNA position. gRNA classes are colored based on sequence abundance as follow: red (1-19 reads), black to green (20-1,000 reads), light-green (>1,000 reads).

The algorithms identified a complete or almost complete set of gRNA classes necessary to edit the mRNAs for the ATPase6 (complex V) and COIII (complex IV) in every strain despite the highly variable number of gRNA classes editing such mRNAs. This clearly shows redundant gRNA classes for some strains. The editing mRNA cascades for ATPase6 and COIII pan-edited genes are shown in **Figure 5** and **Figure S5**, respectively. Aligned gRNAs-mRNAs are provided in the **Supplementary File 2**. Strains PalDa20cl3, Esmeraldo, X109/2 and CANIIIcl1 showed a greater redundancy of gRNA classes than other strains which implied strong variations among and within some DTUs, mainly TcII strains (**Figure 5** and **Figure S5**). On the other side, TcV strains - MNcl2 and LL014R1 - had the lowest redundancy of gRNA classes (**Figure 5** and **Figure S5**).

### Respiratory Complex I: Incomplete Editing Cascades

**Figure 6:**
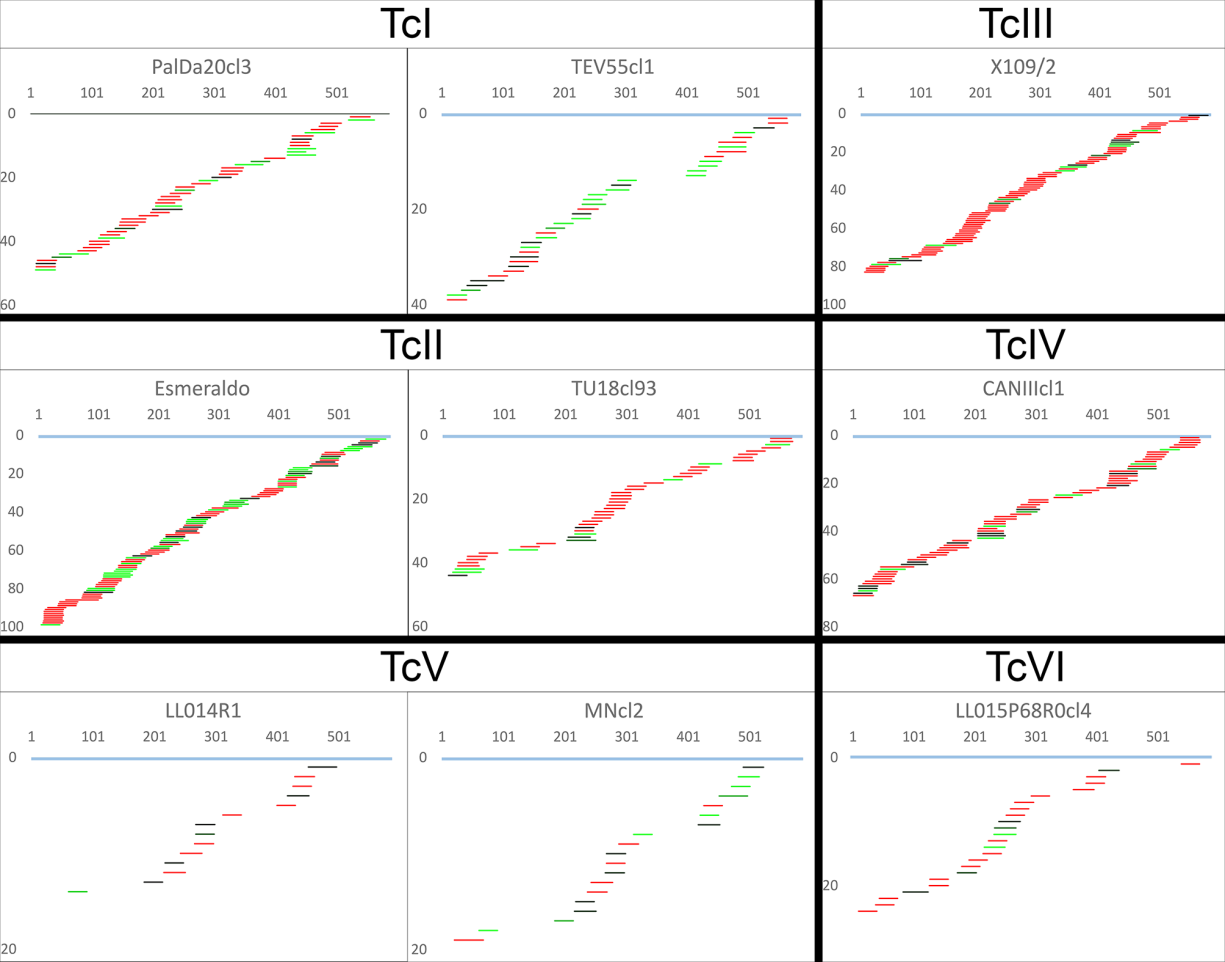
Incomplete editing cascades for NADH dehydrogenase subunit 9. gRNA classes are shown aligned below the fully edited mRNA (light blue). The x-axis indicates mRNA position, and the y-axis indicates the accumulated number of gRNA classes until such mRNA position. gRNA classes are colored based on sequence abundance as follow: red (1-19 reads), black to green (20-1,000 reads), light-green (>1,000 reads).

The percentage of coverage by gRNAs for each mRNA is shown in **Figure 4B** and **Table S5**. Interestingly, the coverage was almost complete for ATPase6 and COIII mRNAs but highly variable for the remaining mRNAs, especially in the complex I members (**Figure 4B**). The editing cascades of the mRNAs coding complex I members —ND9, CR3, CR4, ND3, ND7, ND8— of the respiratory chain are shown in **Figure 6** and **Figures S6–S10**. Even strains showing high mRNA class redundancy in COIII and ATPase6, such as Esmeraldo and X109/1, have incomplete cascades for complex I members. The exception was ND9 mRNA, in which both strains (X109/2 and Esmeraldo) presented almost a complete coverage (**Table S5**), although most of the gRNA classes (82.1% and 74.8%, respectively) had less than 20 reads (see also **Table S6**). Breaking points in ND3, ND7 and ND8 editing cascades in these two strains were also observed. Interestingly, TcV strains —LL014R1 and MNcl2— presented relatively few gRNA classes that edit the complex I components messengers (**Table S3**, **Figure 6** and **Figures S6–S10**) and relatively low coverage of the mRNAs (**Table S5** and **Table S6**).

### Potential Cross-Editing of Mitochondrial mRNAs Among Different DTUs

**Figure 7:**
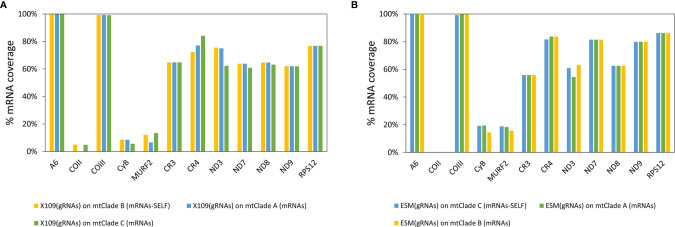
A gRNA repertoire could edit mRNAs from different mitochondrial clades. **(A)** mRNA coverage percentage by X109/2 gRNA repertoire inferred from mRNAs of different mitochondrial clades. **(B)** mRNA coverage percentage by Esmeraldo gRNA repertoire inferred from mRNAs of different mitochondrial clades.

Biparental inheritance of minicircles in hybrid DTUs was previously proposed (Rusman et al., 2019). Here, it was analyzed whether gRNAs from one strain may potentially edit mRNAs from a strain from another DTU, particularly another mitochondrial clade. This ability for “cross-editing” is a requirement in hybrid DTUs, where minicircles come from both parentals but maxicircles come from just one parent. Editing cascades were again generated for Esmeraldo gRNAs and X109/2 gRNAs but based on mRNAs of different mitochondrial clades. mRNA coverage by gRNAs was evaluated and both gRNA repertoires have similar coverages on mRNAs from different mitochondrial clades (**Figure 7**). Cross-editing was also evaluated in more stringent conditions by lowering the allowed number of mismatches in cluster definition (*d* = 3, *d* = 2 and *d* = 1) and it was not detected a significant difference in mRNA coverage percentage. Minor differences were observed in the mRNA coverage percentages for genes CyB and ND9 in *d* = 1 settings (**Figure S11**). These results suggest that biparental inheritance of the kDNA would not generate mRNA editing problems even among phylogenetically distant DTUs.

## Discussion

mRNA editing in kinetoplastids is an intriguing evolutionary mechanism. Questions about how and why such complex and energy-expensive mechanism has evolved are still controversial (Covello and Gray, 1993; Gray et al., 2010; Flegontov et al., 2011). The elucidation of editing cascades in mRNA is a prerequisite to start filling the gaps around such questions. We have previously reported the DNA sequence diversity of the hypervariable region of kinetoplastid minicircles (mHVR) in the main lineages of *T. cruzi*, which were virtually unknown until then (Rusman et al., 2019). Here, we deeply addressed gRNA repertoires (coded in the mHVRs) of nine *T. cruzi* strains belonging to the main linages of this parasite.

Our results showed that gRNA diversity was enormous, with more than 1,300 different gRNA classes, each one detected in >20 reads. Such diversity was structured among the strains according to linkage disequilibrium indexes. However, further analyses are required to address how good the linkage disequilibrium measures perform on this kind of data. In addition, the number of gRNA classes was highly variable among strains. In addition, it was addressed whether shared common secondary structures exist despite the high sequence diversity for gRNAs, as it has been described for *T. brucei* (Schmid et al., 1995). Double-hairpin structures observed in *T. brucei* were not the most frequent structures in *T. cruzi* predictions. However, the predictions should be considered cautiously because they do not include the poly-U commonly added to 3’ of gRNAs (Schmid et al., 1995) and predictions could change if constraints based on experimental data are added to the analysis (Reuter and Mathews, 2010). Finally, despite this study analyzed the mitochondrial editomes of different strains in a comparative and large scale, experimental studies are needed to determine gRNA complete sequences and the abundances of each one. Nevertheless, studies in *T. vivax* and *T. brucei* have revealed that gRNA abundance is related to the coding minicircle abundance (Koslowsky et al., 2014; Greif et al., 2015) although such correlation is not so clear in *Leishmania tarentolae* (Simpson et al., 2015).

As expected from mHVR sequence analyses (Telleria et al., 2006; Rusman et al., 2019), different DTUs shared a relatively low number of gRNA classes, but it was surprising that even strains of the same DTU (here TcI and TcII) shared few gRNA classes. Such strains showed not only different gRNA classes but also very divergent abundances in shared gRNA classes.

The TcI strains analyzed here are phylogenetically close according to nuclear and mitochondrial sequences (Tomasini et al., 2014; Rusman et al., 2020). However, they shared just 19.2% of their gRNA classes. Such results suggest that gRNA repertoires may vary extremely fast. Even TcV strains which are identically at most nuclear analyzed DNA sequences have a poor correlation in the abundances of shared gRNAs, even though TcV strains share most of their mHVR clusters at 95% identity threshold (Rusman et al., 2019) and most of their gRNA classes. Such results suggest that fast changes in minicircle repertoires are not mainly caused by mutation. It is important to mention that the variations in gRNA classes between strains described above would not be a result of minicircle loss after long periods in culture. The strain with more gRNA diversity (Esmeraldo, TcII) was isolated in the eighties and maintained in culture for several years; whereas, for example, LL015P68R0cl4 (TcVI) was isolated ten years ago, cultured for less than a year and then frozen until this study. In addition, LL014R1 (TcV) which showed the lowest number of gRNA classes, was maintained mostly through mouse-triatomine passages since isolation in 2009. In addition, such variations in gRNA class diversity between strains correlate well with mHVR cluster diversity. This result supports that changes in gRNA diversity among strains is not caused by differences in gRNA estimation for different DTUs (caused by edited mRNA prediction).

The models of random or partially random segregation of minicircles (minicircle drift) during kDNA division predicts changes in gRNA composition by variations in gRNA class abundance (Savill and Higgs, 1999). “Minicircle drift” is caused by an error during kDNA network division resulting in the two copies of a replicated minicircle being inherited to the same descendant kinetoplast. Such random changes are analogous to genetic drift in populations (e.g., allele frequency varies randomly until fixation or lost, reducing allele diversity in absence of any other evolutionary force). In the same way, partially random segregation of minicircles predicts loss of gRNA redundancy (Savill and Higgs, 1999). Linkage of gRNA classes (that is, their presence in the same minicircle) may conserve some redundancy. This would occur in the case that redundant gRNA classes were linked with different essential gRNAs (i.e., gRNA classes without redundancy). However, gRNA linkage is still not compatible with spread redundancy observed in some strains because it is expected that the greater the number of redundant classes the greater the number of essential gRNA classes necessary to support them, which strongly limits redundancy.

Since the model of (partial) random segregation as the unique evolutionary force does not fit the high levels of gRNA class redundancy observed in some strains, the genetic exchange would be the most probable force generating gRNA class redundancy. In a previous paper, we proposed biparental inheritance of minicircles in hybrid DTUs of *T. cruzi* (Rusman et al., 2019) and in an intra TcI hybrid (Rusman et al., 2020). Biparental inheritance has also been proposed for *T. brucei* (Turner et al., 1995; Gibson et al., 2008). Maxicircles and minicircles may be biparentally inherited but maxicircles of one parent are lost by drift in few generations (Turner et al., 1995). Despite, minicircles of both parents are maintained by many more generations. In this sense, genetic exchange may generate gRNA redundancy (Savill and Higgs, 1999). In addition, the observed correlated variations in the number of gRNAs classes editing each mRNA (see **Tables 2** and **S4**) may also be explained by a biparental inheritance of minicircles which is a bulky change in gRNA classes. Conversely, clonal propagation would generate a loss of gRNA classes by minicircle drift. Under this hypothetical dynamic, DTUs with less gRNA redundancy (i.e. TcV and TcVI) should have a typical clonal propagation (Lewis et al., 2011; Diosque et al., 2014; Tomasini and Diosque, 2015); while, most gRNA class redundancy has been observed in lineages in which genetic exchange has been reported (TcI, TcII and TcIII) (Llewellyn et al., 2009; Ocana-Mayorga et al., 2010; Baptista Rde et al., 2014; Tomasini and Diosque, 2015; Berry et al., 2019).

Biparental inheritance of minicircles has another challenge, i.e., to make the right editing of mRNA when maxicircles come from just one parent. However, *in silico* analyses showed that gRNA classes from one strain may edit mRNAs from the other mitochondrial clades in a similar way than in the self-clade. This result suggests that genetic exchange and biparental inheritance of minicircles should not cause editing conflicts. In this sense, genetic exchange may increase gRNA class redundancy, avoiding that an editing site being near to lost gRNAs, even restoring broken editing cascades. Consequently, strict clonal lineages would not persist in long evolutionary times (a Muller ratchet).

Interestingly, gRNA class redundancy was mainly detected in ATPase subunit 6 and Cytochrome c oxidase subunit III. Editing cascades of both mRNAs were complete or almost complete in all strains suggesting that such genes are essential for the parasite. However, editing cascades of the complex I subunits have less gRNA class redundancy and even interrupted cascades at several positions in some strains. Functions of the complex I are debatable in trypanosomatids (Opperdoes and Michels, 2008) and it has been proposed that electrons bypass this complex in the respiratory chain of *T. cruzi* epimastigotes (Denicola-Seoane et al., 1992; Carranza et al., 2009). In addition, the absence of two key subunits suggests that if functional for electron transport, the complex I cannot pump protons to intermembranous space and may function to renewing NAD+ (Opperdoes and Michels, 2008) although other studies showed that this activity has no differences between ND mutants and wild type strains in *T. cruzi* epimastigotes (Carranza et al., 2009) although only log-phase epimastigotes were evaluated. Considering the fast evolutionary rates shown for gRNA class frequencies, a completely non-functional complex I should imply highly impaired cascades of gRNA elements editing such sequences. Instead, almost complete editing cascades recovered for some complex I elements do not fit with the hypothesis of non-functional complex I at least in some strains like Esmeraldo or X109 (**Figure 6**). Despite, it is a more suitable hypothesis that time in culture without infecting a mammal allowed that some gRNA classes were lost, or they reduced their abundance as observed. However, it cannot be discarded that some DTUs like TcV may have lost complex I components since the cascades are highly incomplete in both strains in a similar manner. Further studies on this topic are required, especially because deletions in genes coding the complex I subunits were associated with the indeterminate form of the Chagas disease in TcII (Baptista et al., 2006). Nevertheless, our results support that complex I is not required in all stages of the parasite. In this sense, the linkage of different gRNA classes editing different mRNAs may help to protect against gRNA class loss in stages where the complex I is not required.

Another interesting observation was the high frequency of mHVR clusters without gRNA hits. Such “silent” mHVR has been observed in *L. tarentolae* (Simpson et al., 2015) and *T. brucei* (Hajduk et al., 1997) although in minor proportions. There are several potential non-exclusive explanations. Some mHVR clusters may code for gRNAs involved in alternative editing of the mRNAs (which cannot be detected by our current algorithm), or code gRNAs that accumulated mutations or even code for shorter gRNAs (less than 30 bp). Alternatively, many mHVRs may not code for gRNAs and such mHVRs persist by linkage to others that code for essential gRNAs (Savill and Higgs, 2000). However, it is not clear if such situations are enough to explain that around a half of the mHVR clusters did not code for gRNAs in some strains. Interestingly, TcV and TcVI strains, which have relatively less redundancy in gRNA classes, also have relatively less silent mHVR clusters, although the percentage is still not negligible. Consequently, unknown functions of these silent mHVR sequences it is a possibility that cannot be ruled out.

Finally, a model is proposed based on data presented here, from which hypotheses can be derived for further testing. Each minicircle in *T. cruzi* has mHVRs coding for different gRNAs that edit different regions and probably (by random) from different mRNAs (which is also supported by the correlated variations in the number of gRNAs classes editing each mRNA). Such linkage may reduce the chances of losing a gRNA class which is only required in some stages of the lifecycle. Such linkage may also explain the existence of silent mHVRs (although other functions cannot be discarded). In addition, minicircle drift cause loss of gRNA classes and low levels of gRNA class redundancy. Consequently, genetic exchange and biparental inheritance of minicircles may restore gRNA abundances for editing each mRNA site and redundancy which may reduce the chances of lethal loss of essential gRNA classes. The conjunction of minicircle drift and occasional biparental inheritance of kDNA may explain the divergence of gRNA repertoires among strains even within the same DTU.

## Data Availability Statement

The datasets analyzed for this study can be found in the Sequence Read Archive at NCBI with the following accession number PRJNA514922. The source code for gRNA inference is stored at the Open Science Framework https://osf.io/kn34z/.

## Author Contributions

FR: Bioinformatic Analysis, Writing – original draft. NF-Y: Bioinformatic Analysis, Writing – Review and Editing. NT: Conceptualization, Programming, Bioinformatic analysis, Supervision, Writing – Review and Editing. PD: Conceptualization, Funding acquisition, Writing – Review and Editing. All authors contributed to the article and approved the submitted version.

## Funding

The current study is funded by Bunge and Born foundation and the National Scientific and Technical Research Council (D.2555/16. 22920160100063CO) (CONICET, Argentina) to Patricio Diosque.

## Conflict of Interest

The authors declare that the research was conducted in the absence of any commercial or financial relationships that could be construed as a potential conflict of interest.

## Supplementary Material

Supplementary File 1 Zip file containing fasta formatted files with mRNA sequences used for gRNA inference.

Supplementary File 2 Zip file containing gRNA classes aligned to different mRNAs for each strain in this study.

Supplementary File 3
**Tables S1–S6** and **Figures S1–S11**.

Supplementary File 4 Zip file containing predicted gRNA structures in postscript format and the gRNA sequences used for the analysis.
