# Supplementary material for: Guide RNA Repertoires in the Main Lineages of Trypanosoma cruzi: High Diversity and Variable Redundancy Among Strains
Source: Front Cell Infect Microbiol. 2021 May 31;11:663416. doi: 10.3389/fcimb.2021.663416 (PMC8202002; doi:10.3389/fcimb.2021.663416)
Supplement: Supplementary File 3 — Tables S1–S6 and Figures S1–S11 . [file DataSheet_3.pdf]

Supplementary Material

1 Supplementary Figures and Tables

1.1 Supplementary Figures

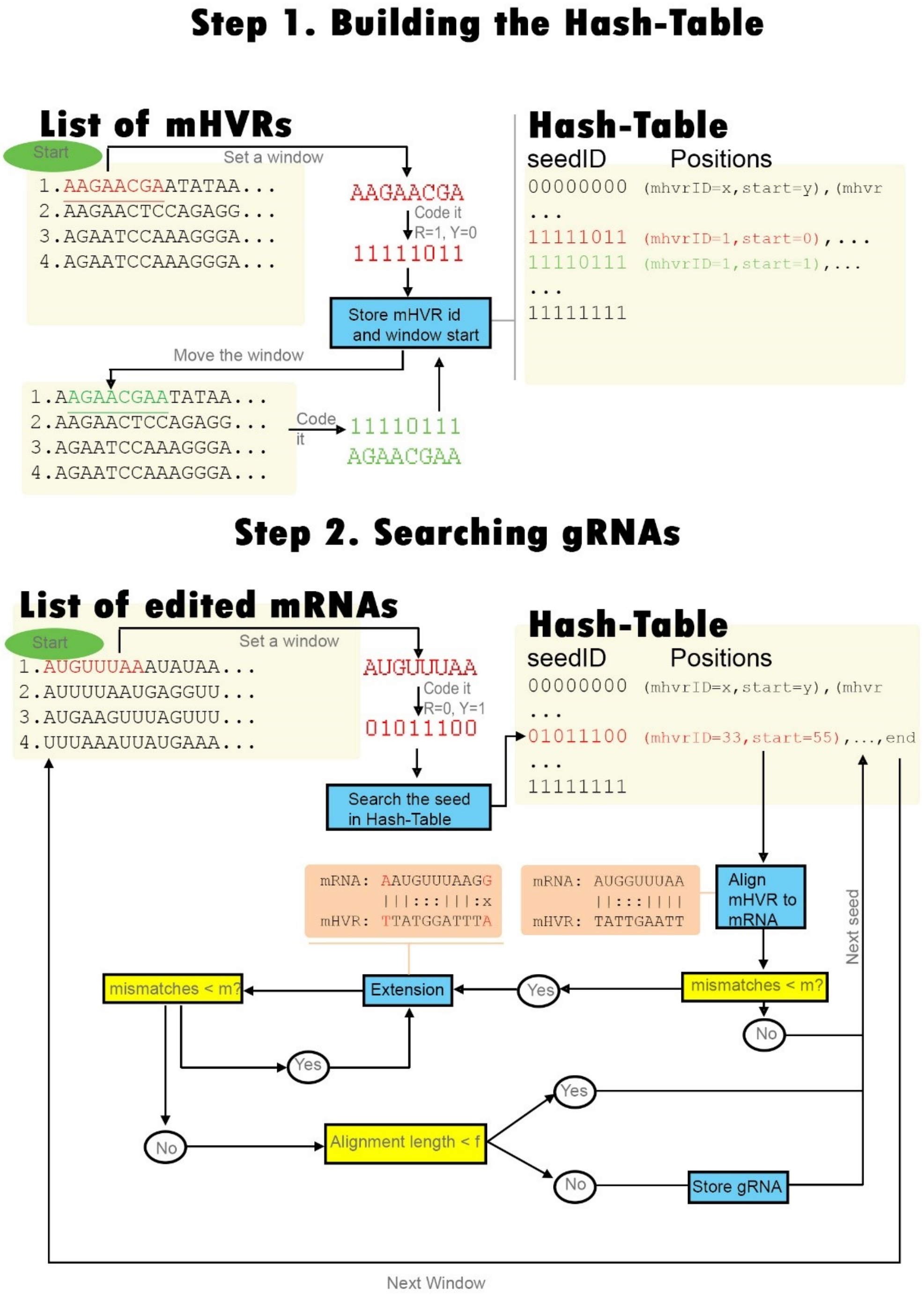

Figure S1. Algorithm scheme for detecting gRNAs on mHVRs (paired-end and merged reads).

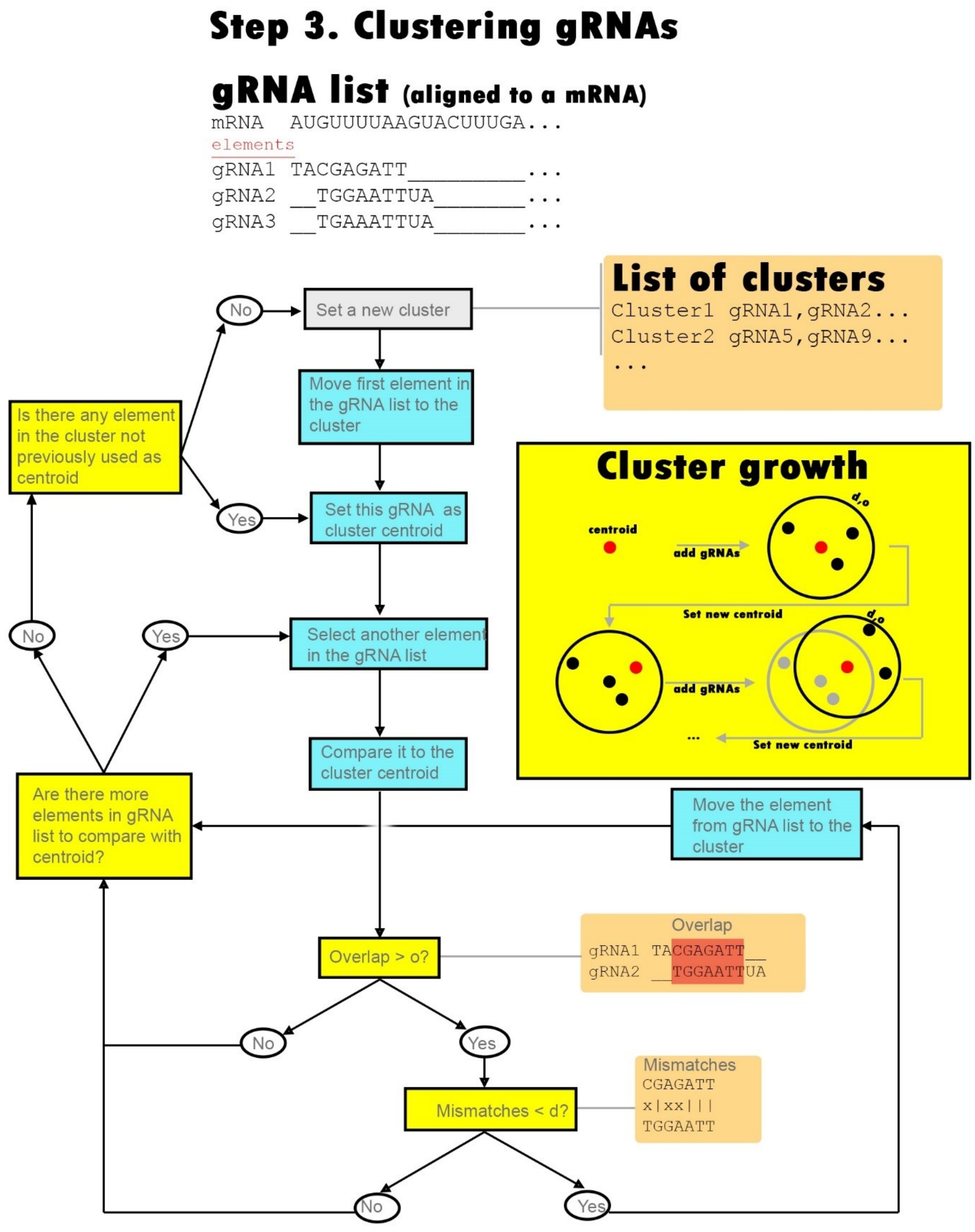

Figure S2. Swarm-like algorithm used to cluster gRNAs according to percentage of sequence overlapping (o) and number of mismatches (d).

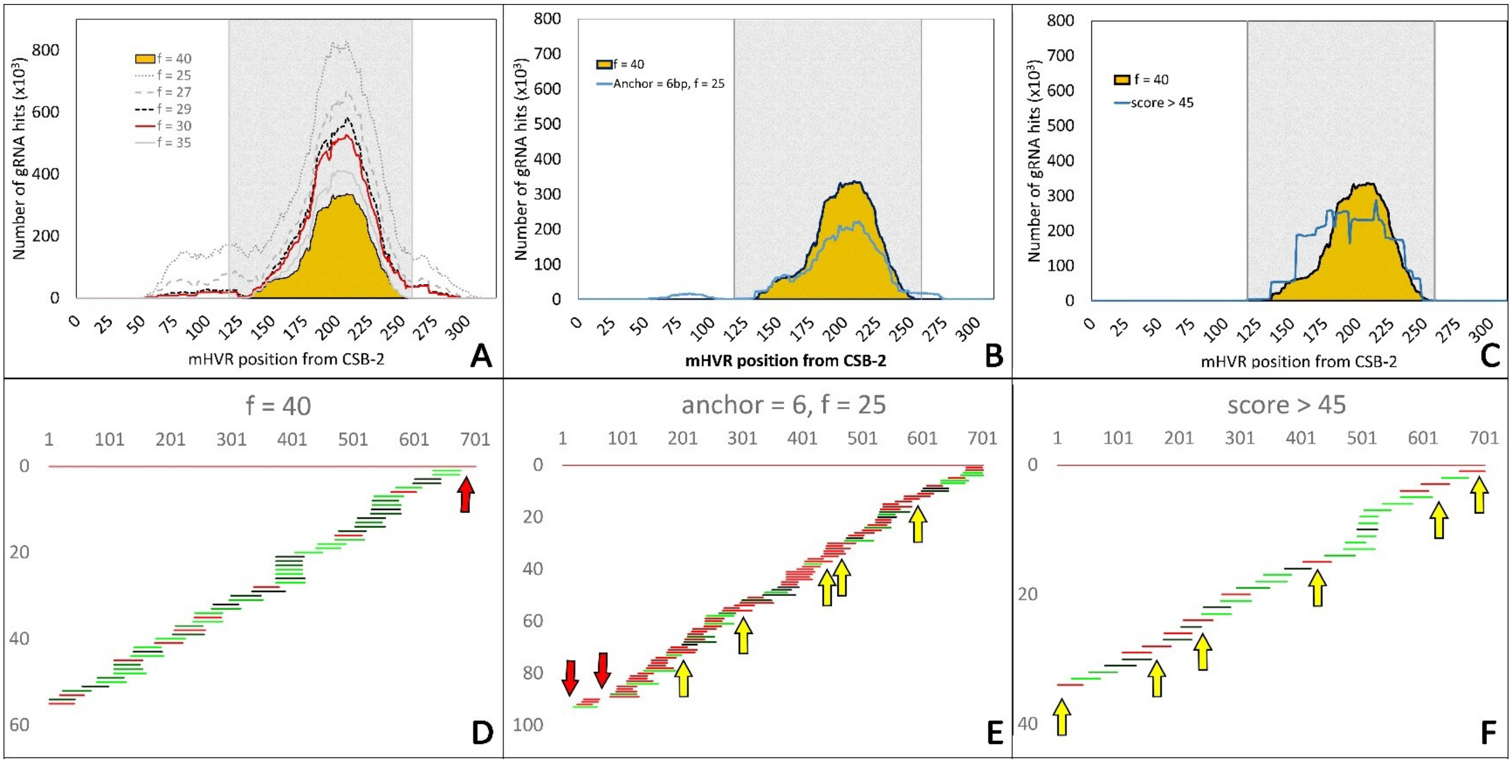

Figure S3. Evaluation of different filters strategies to discard false positive gRNA hits in strain X109/2. A-C, the number of gRNA hits is represented as the number of gRNA bases mapping to a position on the mHVR after applying different filters. The grey-shadow area indicates the gRNA cassette defined by a conservative filter (discarding gRNAs with length <40. i.e., f = 40). Different lines show the applied filter. A, filters only based on the number of bases on the gRNA. B, filter based on the detection of a 6bp anchor and f = 25 (blue line) compared to f = 40. C, filter based on scoring base pairing (canonical =2 and non-canonical =1) and filtering gRNAs with a scoring sum < 45. D-F, editing cascades for ATPase 6 mRNA in strain X109/2 after applying different filters. gRNA classes are shown aligned below the fully edited mRNA (top red line). The x-axis indicates mRNA position, and the y-axis indicates the accumulated number of gRNA classes up to that mRNA position. gRNA classes are colored based on sequence abundance as follow: red (1-19 reads), black to green (20-1000 reads), light-green (>1000 reads). Red arrows indicate interrupted cascade and yellow arrows show regions with low level of coverage (<20 gRNA hits).

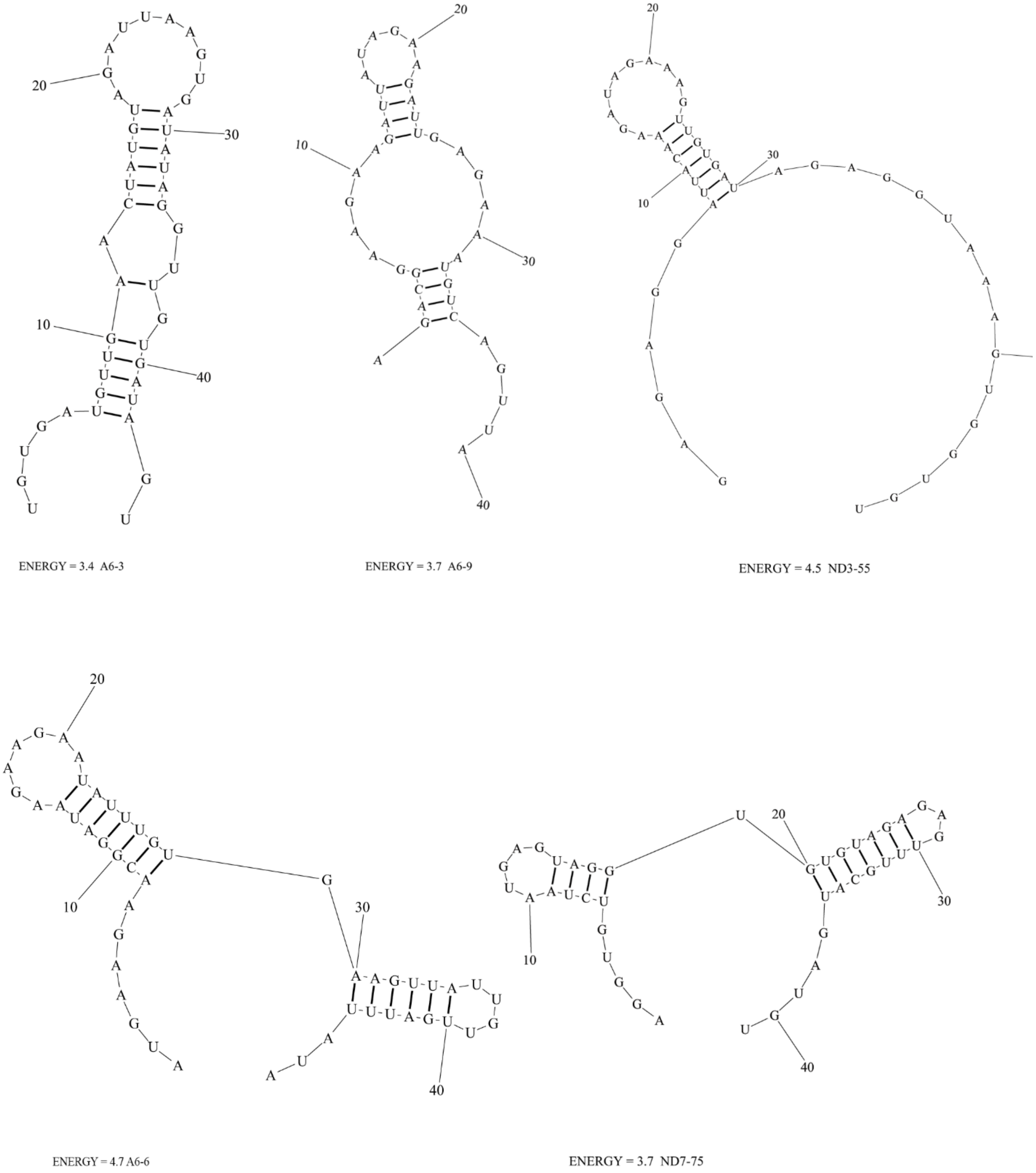

Figure S4. Representative secondary gRNA structures predicted from a subset of 89 gRNAs for all strains analyzed. Stems with two (left), one (mid, more frequent) or no internal loops (or bulges) and ending in a hairpin (figure top). Double hairpins (stem-loops) (figure bottom).

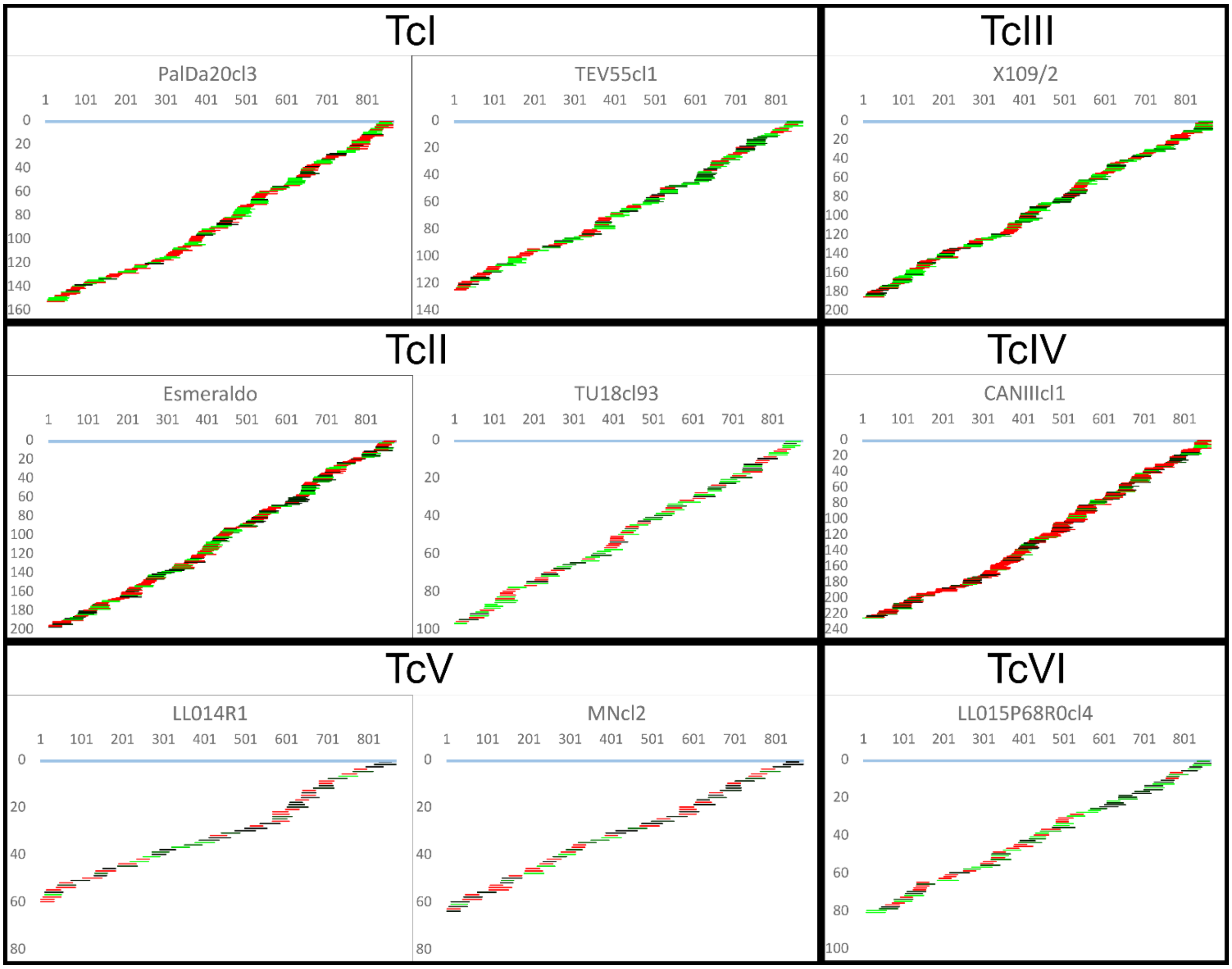

Figure S5. Canonical Cytochrome c oxidase subunit III mRNA editing cascade. gRNA classes are shown aligned below the fully edited mRNA (light blue). The x-axis indicates mRNA position, and the y-axis indicates the accumulated number of gRNA classes until such mRNA position. gRNA classes are colored based on sequence abundance as follow red (1-19 reads), black to green (20-1000 reads), light-green (>1000 reads).

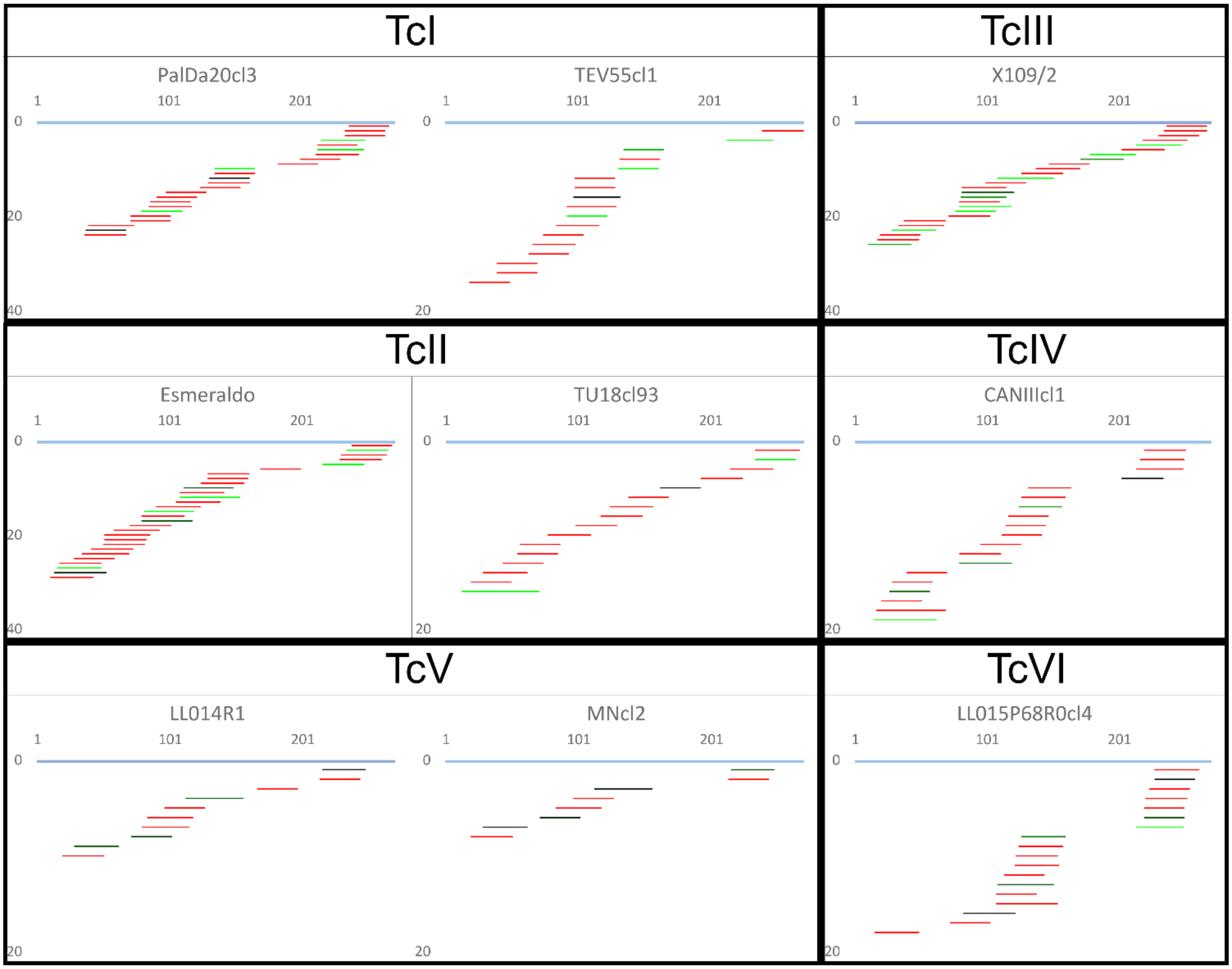

Figure S6. Incomplete editing cascade for NADH dehydrogenase subunit 3 (ND3). Detected gRNA classes are shown aligned below the fully edited mRNA (light blue). The x-axis indicates mRNA position, and the y-axis indicates the accumulated number of gRNA classes until such mRNA position. gRNA classes are colored based on sequence abundance as follow: red (1-19 reads), black to green (20-1000 reads), light green (>1000 reads).

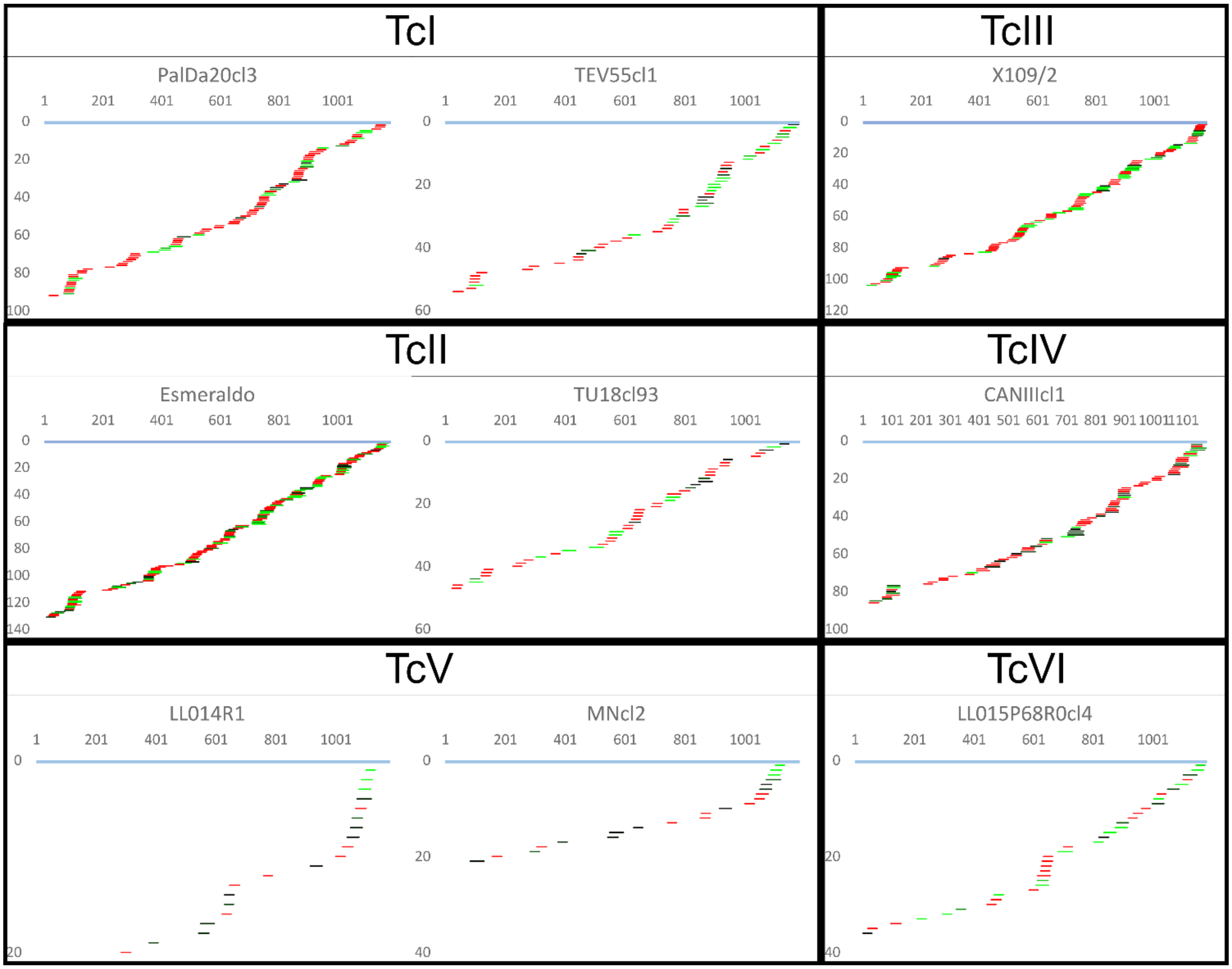

Figure S7. Incomplete editing cascade for NADH dehydrogenase subunit 7 (ND7). Detected gRNA classes are shown aligned below the fully edited mRNA (light blue). The x-axis indicates mRNA position, and the y-axis indicates the accumulated number of gRNA classes until such mRNA position. gRNA classes are colored based on sequence abundance as follow: red (1-19 reads), black to green (20-1000 reads), light-green (>1000 reads).

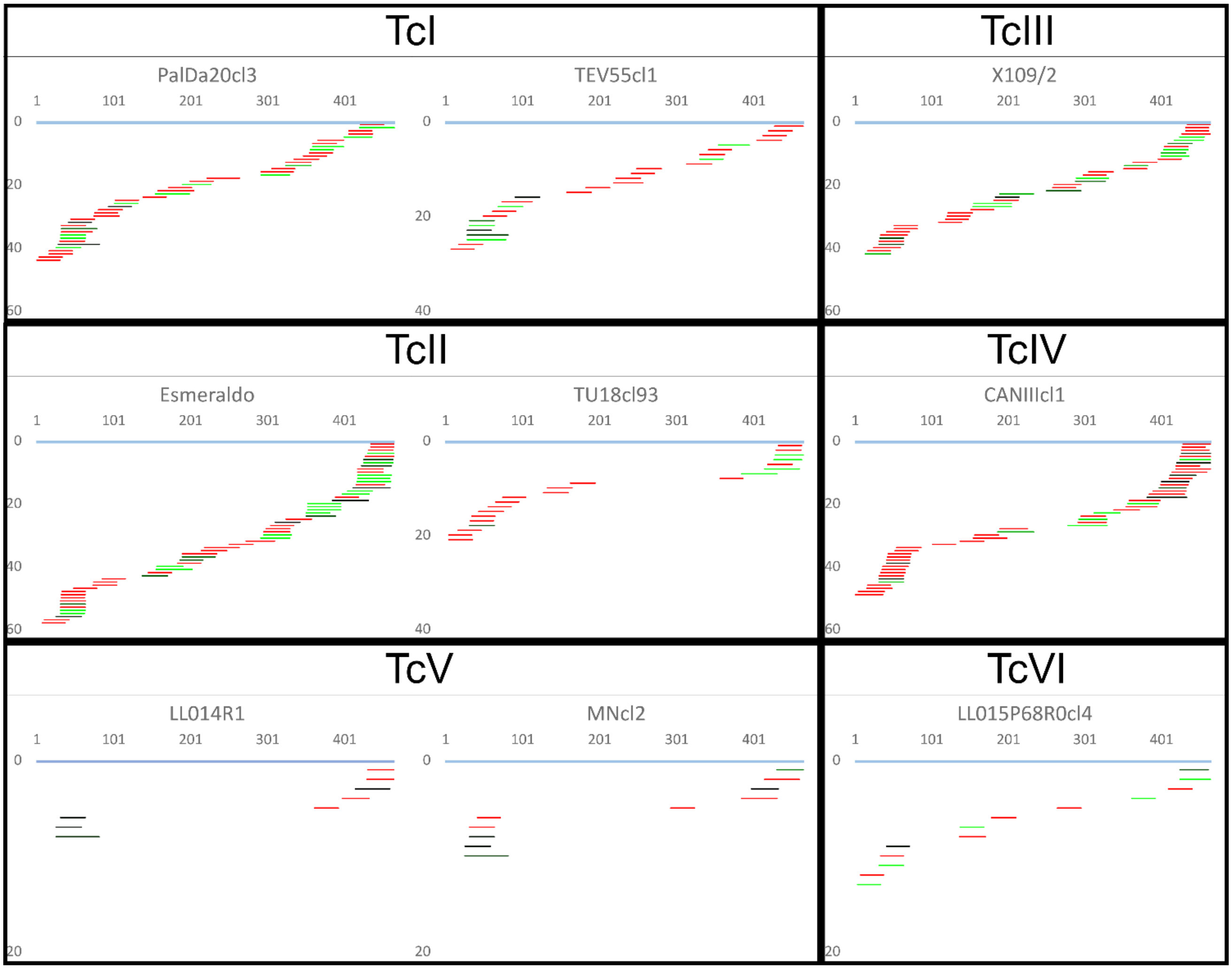

Figure S8. Incomplete editing cascade for NADH dehydrogenase subunit 8 (ND8). Detected gRNA classes are shown aligned below the fully edited mRNA (light blue). The x-axis indicates mRNA position, and the y-axis indicates the accumulated number of gRNA classes until such mRNA position. gRNA classes are colored based on sequence abundance as follow: red (1-19 reads), black to green (20-1000 reads), light-green (>1000 reads).

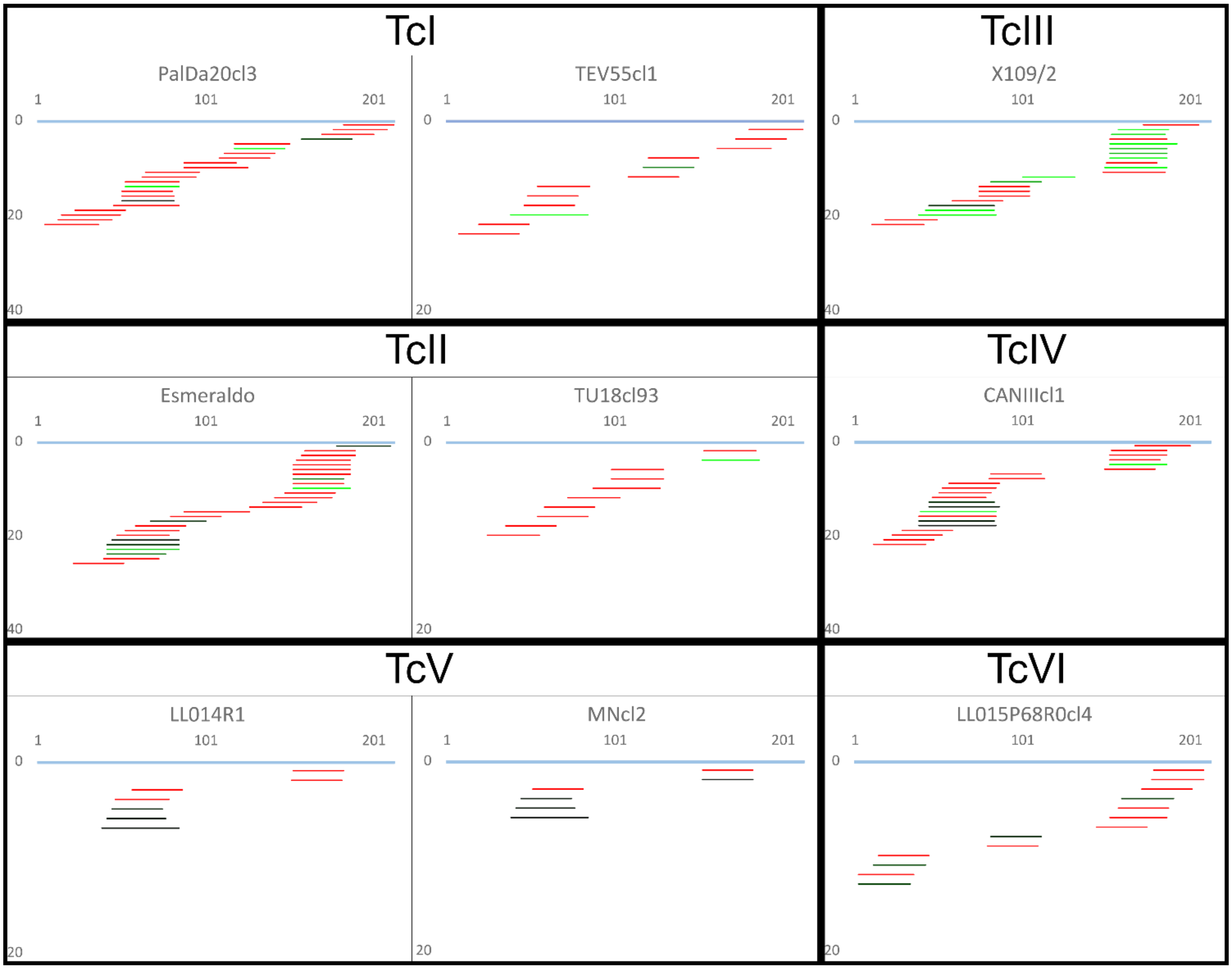

Figure S9. Incomplete editing cascade for CR3. Detected gRNA classes are shown aligned below the fully edited mRNA (light blue). The x-axis indicates mRNA position, and the y-axis indicates the accumulated number of gRNA classes until such mRNA position. gRNA classes are colored based on sequence abundance as follow: red (1-19 reads), black to green (20-1000 reads), light-green (>1000 reads).

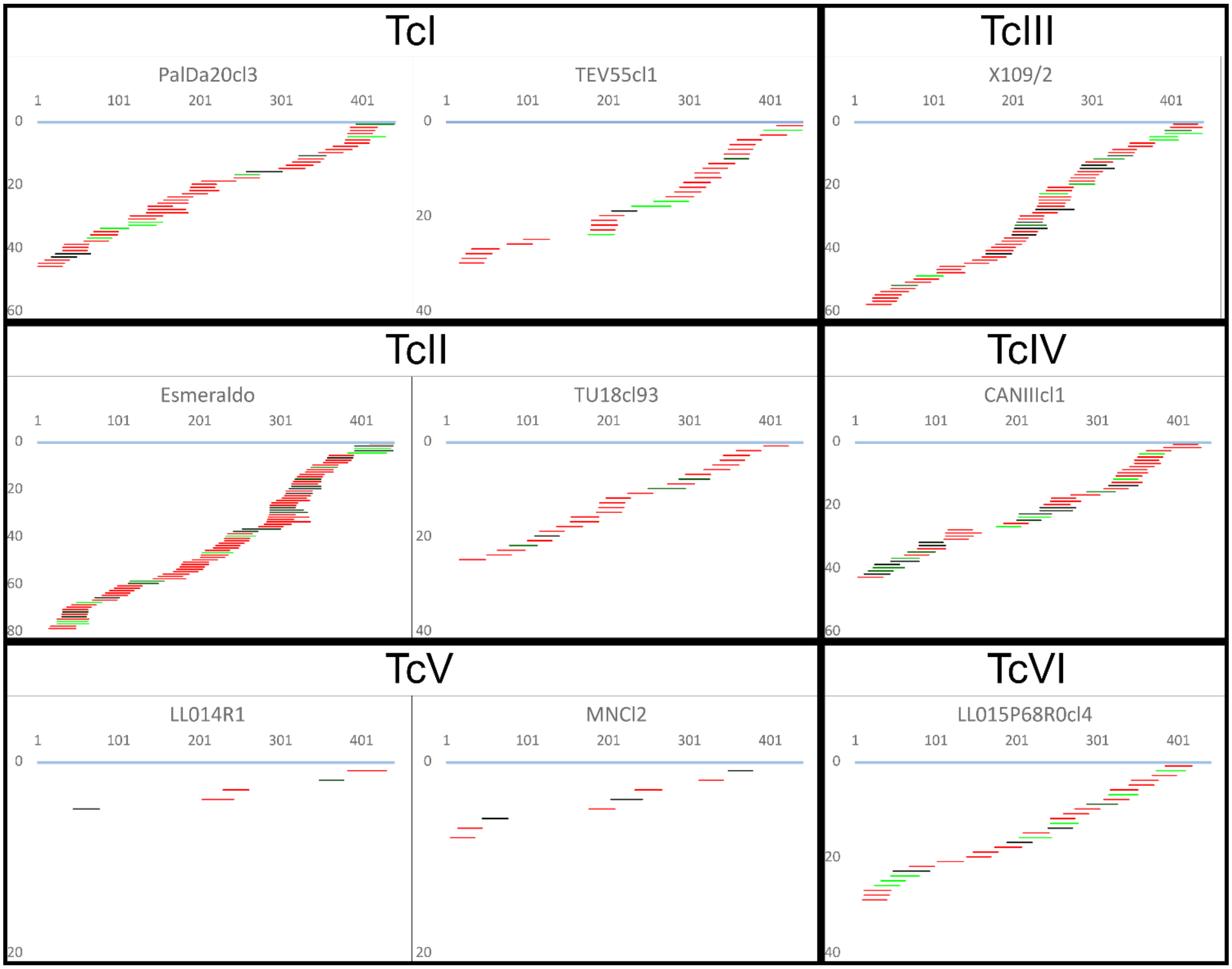

Figure S10. Incomplete editing cascade for CR4. Detected gRNA classes are shown aligned below the fully edited mRNA (light blue). The x-axis indicates mRNA position, and the y-axis indicates the accumulated number of gRNA classes until such mRNA position. gRNA classes are colored based on sequence abundance as follow: red (1-19 reads), black to green (20-1000 reads), light-green (>1000 reads).

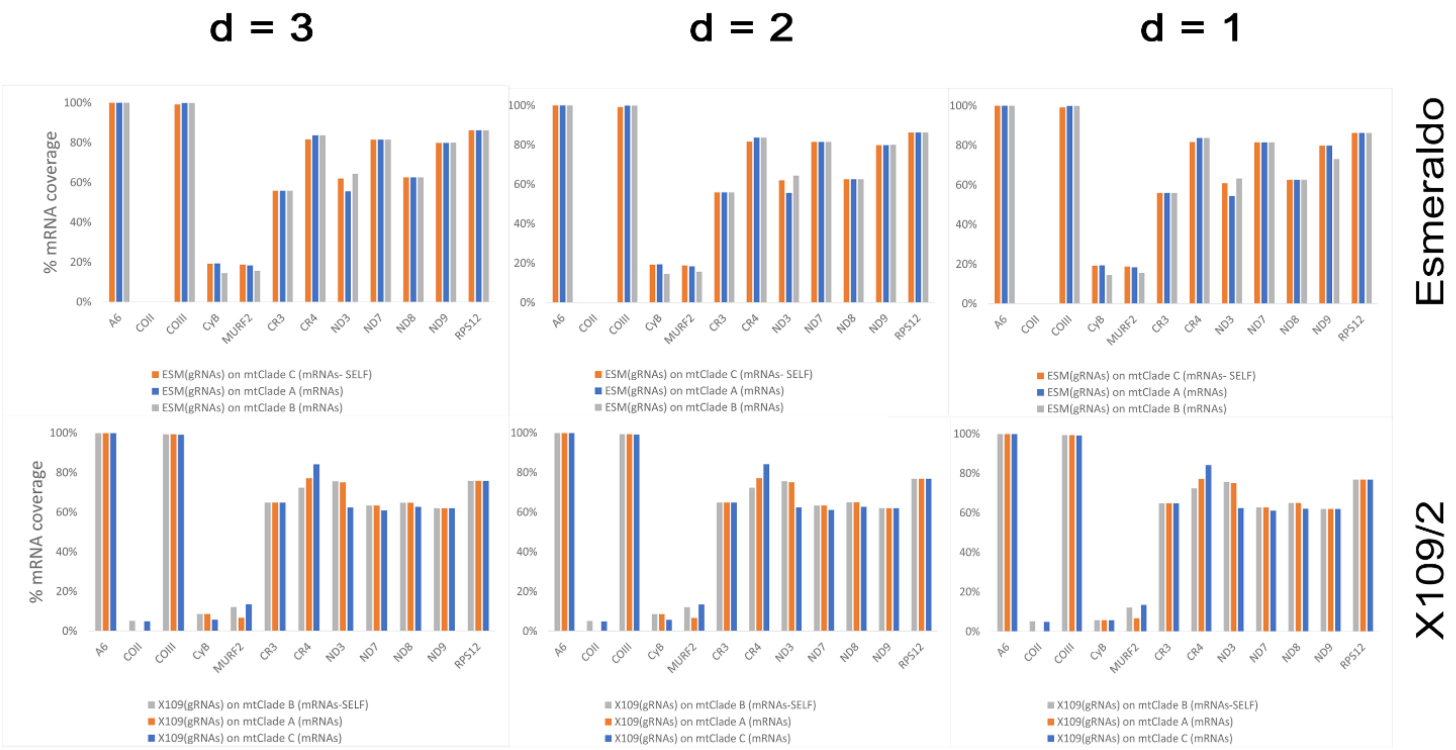

Figure S11. A gRNA repertoire could edit mRNAs from different mitochondrial clades in different stringencies for gRNA class definition parameter (d). (A) mRNA coverage percentage by X109/2 gRNA repertoire inferred from mRNAs of different mitochondrial clades. (B) mRNA coverage percentage by Esmeraldo gRNA repertoire inferred from mRNAs of different mitochondrial clades.

2.2. Supplementary Tables

Table S1. Number of gRNA detected and gRNA classes using predicted mRNAs from other strains* of the same DTU and compared to the references used in this paper.

	**TcI** (mHVRs PalDa20cl3)		**TcII** (mHVRs Esmeraldo)		**TcIII** (mHVRs X109/2)	
	mRNAs from Colombiana	mRNAs from Sylvio	mRNAs from S23b	mRNAs from Esmeraldo	mRNAs from 231	mRNAs from CL-Brener
gRNA hits	1,219,969	1,214,475	679,744	693,075	777,688	801,591
gRNA classes	236	234	402	401	326	319

*Sequences were obtained from Reis-Cunha et al. (41). Strains Colombiana(TcI), S23b(TcII) and 231(TcIII) were analyzed since they have highest pairwise distance to Sylvio, Esmeraldo and CL Brener, respectively.

Table S2. Number of mHVR clusters in different states for coding gRNAs

mHVR clusters with gRNA hits in:	PalDa	TEV	TU18	ESM	X109	CAN	MN	LL014	LL015
>75% reads	156	136	89	195	207	64	31	39	75
<10% reads	164	96	60	151	164	83	25	9	32
>10% & < 75%	4	2	2	1	2	2	15	24	1

Table S3. Number of gRNA classes detected for each mRNA.

	ESM	TU18	PALDA	TEV	X109	CAN	MN	LL014	LL015
A6	102	34	42	38	63	54	26	23	44
COII*	0	1	1	2	1	2	1	1	1
COIII	114	61	85	82	118	95	38	37	55
CyB*	10	3	7	1	3	2	3	0	2
MURF2*	7	6	7	2	4	5	4	4	3
CR3	8	1	4	2	12	5	3	3	4
CR4	25	4	11	6	18	19	3	2	10
ND3	8	3	6	5	10	5	4	4	6
ND7	60	17	28	26	40	33	13	15	20
ND8	26	5	16	9	17	15	5	4	7
ND9	26	11	19	10	15	20	8	6	7
RPS12	15	10	8	12	18	12	7	5	6

*partially edited

Table S4. Correlation coefficient between the number of gRNAs editing a particular (pan-edited) mRNA and the number of gRNAs editing a another different (pan-editied) mRNA. Each value is calculated comparing gRNA classes for each mRNA pairs across difrent strains.

	COIII	CR3	CR4	ND3	ND7	ND8	ND9	RPS12
A6	0.82	0.70	0.93	0.73	0.98	0.93	0.84	0.72
COIII		0.75	0.88	0.79	0.89	0.89	0.82	0.91
CR3			0.76	0.95	0.73	0.71	0.51	0.75
CR4				0.76	0.93	0.94	0.87	0.73
ND3					0.76	0.76	0.51	0.73
ND7						0.97	0.87	0.78
ND8							0.94	0.72
ND9								0.63

Table S5. Percentage of coverage of mRNA sequence by different gRNA classes

	PalDa	TEV	TU18	ESM	X109	CAN	MN	LL014	LL015
A6	100%	100%	98%	100%	100%	100%	100%	100%	99%
COII*	55%	37%	37%	41%	34%	42%	18%	10%	21%
COIII	100%	100%	100%	100%	100%	100%	100%	100%	99%
CyB*	55%	41%	43%	53%	58%	31%	21%	17%	34%
MURF2*	64%	59%	54%	71%	77%	47%	24%	25%	35%
CR3	95%	82%	66%	89%	85%	73%	37%	38%	66%
CR4	100%	84%	91%	97%	96%	93%	51%	39%	91%
ND3	79%	76%	94%	86%	94%	70%	60%	71%	63%
ND7	85%	71%	73%	92%	87%	83%	42%	36%	62%
ND8	93%	82%	60%	94%	85%	86%	37%	34%	55%
ND9	92%	81%	87%	98%	98%	94%	57%	47%	68%
RPS12	93%	98%	86%	88%	94%	90%	74%	74%	91%

*partially edited

Table S6. Percentage of coverage of mRNA sequence by different gRNA classes (> 20 gRNAs per gRNA class)

	PalDa	TEV	TU18	ESM	X109	CAN	MN	LL014	LL015
A6	99%	100%	96%	100%	100%	97%	87%	87%	99%
COII	5%	10%	6%	0%	5%	8%	5%	5%	5%
COIII	99%	99%	100%	100%	99%	98%	98%	97%	99%
CyB	16%	3%	8%	19%	9%	6%	9%	0%	5%
MURF2	21%	6%	18%	19%	12%	14%	12%	10%	9%
CR3	46%	37%	16%	56%	65%	39%	31%	22%	49%
CR4	66%	46%	32%	82%	72%	67%	24%	15%	59%
ND3	50%	40%	44%	61%	75%	56%	52%	52%	45%
ND7	53%	45%	44%	81%	64%	55%	27%	28%	45%
ND8	74%	34%	25%	63%	65%	57%	27%	22%	37%
ND9	76%	44%	45%	80%	62%	67%	33%	35%	29%
RPS12	72%	88%	77%	86%	77%	75%	64%	57%	69%
