# Supplementary figures and images for: Guide RNA Repertoires in the Main Lineages of Trypanosoma cruzi: High Diversity and Variable Redundancy Among Strains
Source: Front Cell Infect Microbiol. 2021 May 31;11:663416. doi: 10.3389/fcimb.2021.663416 (PMC8202002; doi:10.3389/fcimb.2021.663416)

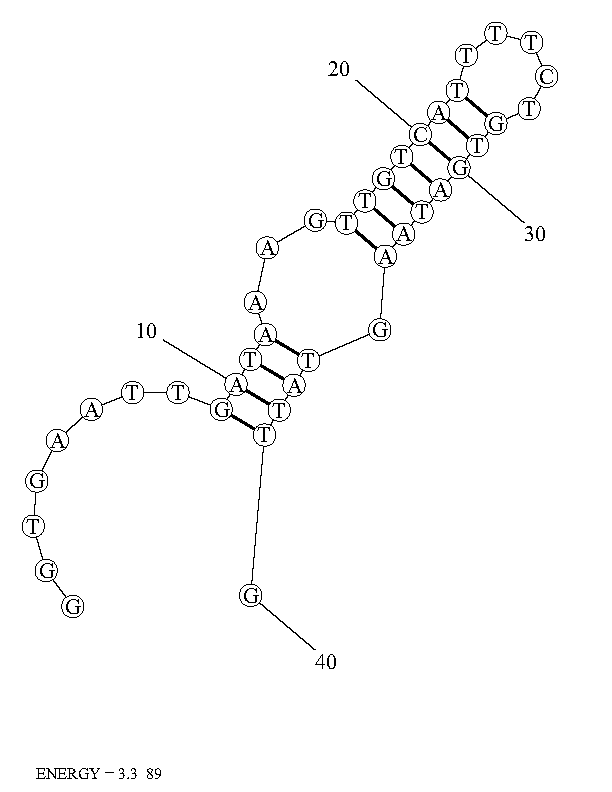

Supplement: Supplementary File 4 — Zip file containing predicted gRNA structures in postscript format and the gRNA sequences used for the analysis. [file DataSheet_4.zip › Supplementary file 4/Animation.gif]
